# Adaptive regulation of miRNAs/milRNAs in tissue-specific interaction between apple and *Valsa mali*

**DOI:** 10.1093/hr/uhae094

**Published:** 2024-04-02

**Authors:** Chengyu Gao, Binsen Zhao, Jian Zhang, Xuan Du, Jie Wang, Yan Guo, Yanting He, Hao Feng, Lili Huang

**Affiliations:** State Key Laboratory for Crop Stress Resistance and High-Efficiency Production, College of Plant Protection, Northwest A&F University, Yangling, Shaanxi 712100, China; State Key Laboratory for Crop Stress Resistance and High-Efficiency Production, College of Plant Protection, Northwest A&F University, Yangling, Shaanxi 712100, China; State Key Laboratory for Crop Stress Resistance and High-Efficiency Production, College of Plant Protection, Northwest A&F University, Yangling, Shaanxi 712100, China; State Key Laboratory for Crop Stress Resistance and High-Efficiency Production, College of Plant Protection, Northwest A&F University, Yangling, Shaanxi 712100, China; State Key Laboratory for Crop Stress Resistance and High-Efficiency Production, College of Plant Protection, Northwest A&F University, Yangling, Shaanxi 712100, China; State Key Laboratory for Crop Stress Resistance and High-Efficiency Production, College of Plant Protection, Northwest A&F University, Yangling, Shaanxi 712100, China; State Key Laboratory for Crop Stress Resistance and High-Efficiency Production, College of Plant Protection, Northwest A&F University, Yangling, Shaanxi 712100, China; State Key Laboratory for Crop Stress Resistance and High-Efficiency Production, College of Plant Protection, Northwest A&F University, Yangling, Shaanxi 712100, China; State Key Laboratory for Crop Stress Resistance and High-Efficiency Production, College of Plant Protection, Northwest A&F University, Yangling, Shaanxi 712100, China

## Abstract

In plant-pathogen interactions, pathogens display tissue specificity, infecting and causing disease in particular tissues. However, the involvement of microRNAs/microRNA-like RNAs (miRNAs/milRNAs) in tissue-specific regulation during plant-pathogen interactions remains largely unexplored. This study investigates the differential expression of miRNAs/milRNAs, as well as their corresponding target genes, in interactions between *Valsa mali* (*Vm*) and different apple tissues. The results demonstrated that both apple miRNAs and *Vm* milRNAs exhibited distinct expression profiles when *Vm* infected bark and leaves, with functionally diverse corresponding target genes. Furthermore, one apple miRNA (Mdo-miR482a) and one *Vm* milRNA (Vm-milR57) were identified as exhibiting tissue-specific expression in interactions between *Vm* and apple bark or leaves. Mdo-miR482a was exclusively up-regulated in response to *Vm* infection in bark and target a nucleotide-binding leucine-rich repeat (NLR) gene of apple. When Mdo-miR482a was transiently over-expressed or silenced, the resistance was significantly reduced or improved. Similarly, transient expression of the NLR gene also showed an increase in resistance. Vm-milR57 could target two essential pathogenicity-related genes of *Vm*. During *Vm* infection in bark, the expression of Vm-milR57 was down-regulated to enhance the expression of the corresponding target gene to improve the pathogenicity. The study is the first to reveal tissue-specific characteristics of apple miRNAs and *Vm* milRNAs in interactions between *Vm* and different apple tissues, providing new insights into adaptive regulation in tissue-specific interactions between plants and fungi.

## Introduction

Phytopathogens frequently target and thrive on specific plant organs and tissues, such as leaf, stem, flower, root, xylem, phloem, mesophyll, or particular developmental stages of their hosts [1]. Some pathogens display a high degree of structural specificity, like powdery mildew that primarily infects the leaf epidermis, while others, such as *Sclerotinia sclerotiorum*, exhibit a lack of structural specificity and can infect a wide range of tissues [2]. Recent studies have highlighted the importance of adaptations in diverse plant pathogens, including bacteria, fungi, and oomycetes, for efficient invasion and colonization of specific host tissues [1, 3–5]. For example, *Phytophthora palmivora* has the ability to infect the roots of barley plants but does not typically infect the leaf epidermis unless there is prior leaf damage. This distinction is likely attributed to the absence of cuticles in the roots [6]. *Arabidopsis thaliana*, affected by the downy mildew pathogen *Hyaloperonospora arabidopsidis*, commonly experiences leaf infections. These infections induce different defense responses against *H. arabidopsidis*, as a result of the varied activation of R genes [7]. *Ustilago maydis* can infect multiple maize tissues, with effector proteins influencing pathogenicity for distinct organs, suggesting that individual fungal effector proteins can play a role in organ-specific fungal pathogenicity [8]. However, research on the mechanisms underlying pathogen-host specificity is still in its early stage, and many questions remain partially answered.

Small RNAs (sRNAs), encompassing small interfering RNAs (siRNAs), microRNAs (miRNAs), and Piwi-interacting RNAs (piRNAs), are prevalent in a diverse range of eukaryotic organisms [9]. The generation of miRNAs and siRNAs from their precursor molecules is catalyzed by RNase III-like enzymes, specifically enzymes like Drosha, Dicer, or DICER-LIKE (DCL) proteins, whereas the biogenesis of piRNAs occurs independently of Dicer [10]. These sRNAs engage with Argonaute proteins to assemble RNA-induced silencing complexes (RISCs). The RISCs possess the capacity to either directly cleave target RNAs or recruit additional proteins to the target RNAs and their associated chromatin, ultimately initiating post-transcriptional or transcriptional gene silencing [11].

sRNAs serve as key regulators in plant-pathogen interactions [12]. On one hand, plant sRNAs participate in plant immunity. For instance, miR393 in Arabidopsis is triggered by a PAMP, flg22, which leads to the silencing of auxin receptors. This downregulation of the auxin signaling pathway subsequently activates the pattern-triggered immunity (PTI) response [13]. Through modulating auxin homeostasis, rice siR109944 negatively regulates plant immunity against sheath blight and exerts impacts on multiple agronomic traits [14]. On the other hand, pathogens utilize endogenous sRNAs to regulate their infection. *Phytophthora* sRNAs are associated with RxLR and Crinkler effectors and may regulate them, impacting pathogen pathogenicity [15, 16]. milR-87, a fungal microRNA-like RNA (milRNA) which exists in *Fusarium oxysporum* f. sp. *cubense* and possess features of animal and plant miRNAs, can enhance pathogen pathogenicity by suppressing the expression of a glycosyl hydrolase coding gene [17]. Additionally, certain sRNAs possess the ability to traverse between hosts and interacting microbes/parasites, facilitating the trans-silencing of target genes belonging to their respective counterparts. *Arabidopsis* delivers sRNAs into the fungus *Botrytis cinerea* via exosome-like extracellular vesicles (EVs), and the transferred host sRNAs affect fungal pathogenicity [18]. A novel milRNA from *Puccinia striiformisf*. sp. *tritici* (*Pst*) has the capability to suppress wheat defenses by silencing wheat pathogenesis-related 2 (PR2) [19].

Previous studies have proven that sRNA expression in plants often exhibits spatial and temporal specificity [20]. Plant miRNAs in *Medicago truncatula* can be enriched in specific tissues, such as roots [21]. Numerous organ-specific miRNAs have been discovered in *Arabidopsis*, with a dominant portion highly expressed in flowers [22]. sRNAs in maize also display tissue specificity and evolutionary dynamics [23]. Meanwhile, fungal milRNAs in *F. oxysporum* [24], *Coprinopsis cinerea* [25], and *Volvariella volvacea* [26] exhibit tissue-specific expression. However, it remains largely unknown whether sRNA expression in plants and fungi differs when pathogens interact with different host tissues or whether sRNAs are involved in regulating tissue-specific pathogen infections.

Apple Valsa canker, a destructive apple (*Malus domestica*) disease caused by the fungus *Valsa mali*, leads to yield losses in eastern Asia annually and threatens apple production safety [27, 28]. Pathogen infestation primarily occurs through wounds or natural ostioles on the bark [29]. The pathogenic fungi’s mycelium can extensively invade healthy apple bark, phloem, and xylem tissues, ultimately causing severe host tissue necrosis [28, 30]. Although *V. mali* only infects bark in the field, it can cause lesions on leaves under artificial injury-induced inoculation conditions [31]. Notably, *V. mali* could be detected in the asymptomatic apple leaves (data not shown), implying it can infect leaf tissues but not cause disease. Consequently, the difference of colonization and expansion mechanism of *V. mali* in different apple tissues may lead to the tissue-specific pathogenesis.

To address the knowledge gap regarding how miRNAs and milRNAs exhibit differential expression patterns and functions during pathogen infecting different host tissues, we utilized the *V. mali*-apple interaction system in this study. We selected bark and leaf samples artificially inoculated with *V. mali in vitro* for small RNA sequencing (sRNA-Seq). By analysing sRNA-Seq data, we mapped the expression profiles of tissue-specific miRNAs in hosts and pathogens and investigated their potential regulatory networks. Based on this analysis, we identified two key sRNAs, one in apple and another in *V. mali*, and further validated their tissue-specific functions in host-fungi interactions. Our study demonstrates that sRNAs play a role in regulating pathogen-host tissue-specific interactions, providing a theoretical foundation for exploring the mechanisms underlying pathogen preferences for infecting specific host tissues.

## Results

### Small RNA profiles and identification of novel miRNAs in *M. domestica*

To investigate the expression profiles of apple miRNAs during fungal infection and to discern whether host miRNAs exhibit distinct expression patterns when *V. mali* infects different tissues, a total of 12 small RNA libraries were prepared and sequenced from four sample types: healthy apple bark and leaves (BMd and LMd), the junction of healthy and diseased apple bark inoculated with *V. mali* at 24 hours post-inoculation (hpi) (IBMd), and the junction of healthy and diseased apple leaves inoculated with *V. mali* at 24 hpi (ILMd). Each sample type included three biological replicates. Comprehensive information pertaining to the sequencing results can be found in Table S1 (see online supplementary material).

After filtering the sequencing data, we further eliminated known apple miRNAs [32] to obtain valid data for the prediction of novel miRNAs. As a result, we identified 678 novel miRNAs, each possessing unique precursors as evidenced by self-blasting of precursors. To distinguish them from known apple miRNAs, the novel miRNAs were designated as Md-miRN1 (Md-miR_novel1) through Md-miRN678 (Table S2, see online supplementary material).

To characterize the novel miRNAs, we counted their lengths distribution and first nucleotide bias. The novel apple miRNAs exhibited a length distribution ranging from 20 to 24 nucleotides (nt), with 24-nt miRNAs representing the majority at 86.6%, followed by 21-nt miRNAs accounting for 9.7% of the total (Fig. S1A, see online supplementary material). This length range aligned with the typical features of plant miRNAs [33]. Examination of the first nucleotide composition of mature miRNAs revealed that the majority of 21 nt-long miRNAs possessed 5′-uridine residues, suggesting that the 21 nt-long novel miRNAs are in line with the majority of previously reported plant miRNA profiles [34] (Fig. S1B, see online supplementary material).

### Analysis of differentially expressed miRNAs and potential miRNA targets in different tissues of apple under infectious conditions

**Figure 1:**
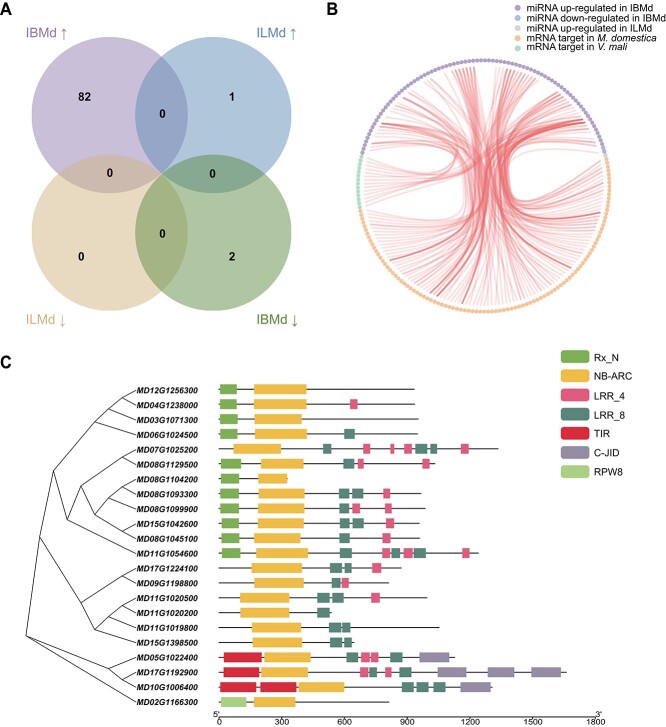
Profile of tsDEMs of *Malus domestica* and the structures of their corresponding target genes when *Valsa mali* infected different tissues of host. (**A**) Venn diagram showing the number of up-regulated (up arrows) and down-regulated (down arrows) apple miRNAs in apple bark and leaves inoculated with *V. mali* (IBMd and ILMd), respectively, at 24 h. (**B**) Network diagram showing the interaction between apple tsDEMs and their endogenous and exogenous target genes. (**C**) Evolutionary relationships and functional domain structures of NLR genes, which were targeted by upregulated tsDEMs in IBMd.

To examine tissue-specific transcriptional changes in microRNA (miRNA) during apple-*Vm* interactions, we aligned sequencing data to 945 known and novel apple miRNAs for quantification (Table S2, see online supplementary material). We conducted differential expression analysis of miRNAs in bark and leaves during various stages of pathogen infection, using BMd and LMd samples as controls. Interestingly, we observed substantial differences in the quantity of DEMs in both bark and leaves. A total of 84 miRNAs exhibited differential expression in IBMd, while only one miRNA displayed differential expression in ILMd (Fig. 1A; Table S3, see online supplementary material). There was no overlap between the two groups. We designated DEMs specifically expressed in distinct tissues as tissue-specific differentially expressed miRNAs (tsDEMs). Ultimately, we identified 84 tsDEMs (82 up-regulated and two down-regulated) in IBMd and only one up-regulated tsDEM in ILMd (Fig. 1A). These findings revealed that the endogenous miRNA expression patterns in different host tissues were entirely distinct upon pathogen infection. *V. mali* infestation could not elicit significant alterations in apple leaf miRNA expression.

To identify the targets of tsDEMs, we employed *in silico* and degradome analyses to predict endogenous (in *V. mali*) and exogenous (in *M. domestica*) targets of apple miRNAs. Specifically, we disregarded exogenous targets for down-regulated miRNAs. By predicting the targets of 85 tsDEMs, we identified a total of 116 targets (102 in *M. domestica* and 14 in *V. mali*) in *M. domestica* and *V. mali* for 59 miRNAs and the remaining 26 miRNAs have no targets. (Table S4, see online supplementary material). Among these miRNAs, 36 targeted multiple genes, and 23 targeted only a single gene. Of the 36 miRNAs capable of targeting multiple genes, six miRNAs targeted both endogenous and exogenous genes. Notably, the sole tsDEM in ILMd did not exhibit targets in either the pathogen or the host (Fig. 1B).

### Functional analysis for targets of apple tsDEMs

To elucidate the functions of target genes modulated by tsDEMs, structural domain prediction and functional annotation for all candidate targets were conducted. Remarkably, only two miRNAs exhibited down-regulation in IBMd. Md-miRN261, targeting MD07G1308200, a gene in *M. domestica*, harbored the NDUFA12 domain (Table S4, see online supplementary material). In contrast, Md-miRN493 lacked a target gene in *M. domestica*. It is hypothesized that miRNAs in bark may positively influence target gene expression during the infection phase by down-regulating their expression. Functional annotations of target genes in *M. domestica*, governed by up-regulated miRNAs in IBMd, revealed that these genes primarily belong to transcription factors (TFs), disease resistance genes, cellular enzymes like kinases and growth-regulating factor (Table S4, see online supplementary material). Intriguingly, we observed that several targets shared the NB-ARC domain (Fig. 1C). Most plant resistance (R) proteins, also referred to as NLR, possess the NB-ARC domain, which acts as a molecular switch, modulating NLR activation through nucleotide binding and hydrolysis [35]. This result suggested that the majority of targets (21.6%) were associated with NLR genes, and miRNAs may negatively regulate host NLR genes by up-regulating their expression in bark, but not in leaves. Furthermore, we identified that upregulated tsDEMs in IBMd also influenced pathogen target genes with diverse functions, such as ABC transporter protein, enzymes like hydrolase, TFs like HLH, and ribosomal protein (Table S4, see online supplementary material). This indicated that, when apple bark was challenged with *V. mali*, the host may counteract pathogen transport, transcription, enzymatic activity, and ribosomal function by up-regulating miRNA expression.

### A tissue-specific miRNA Mdo-miR482a in apple could reduce the resistance of apple by targeting a host NLR gene

**Figure 2:**
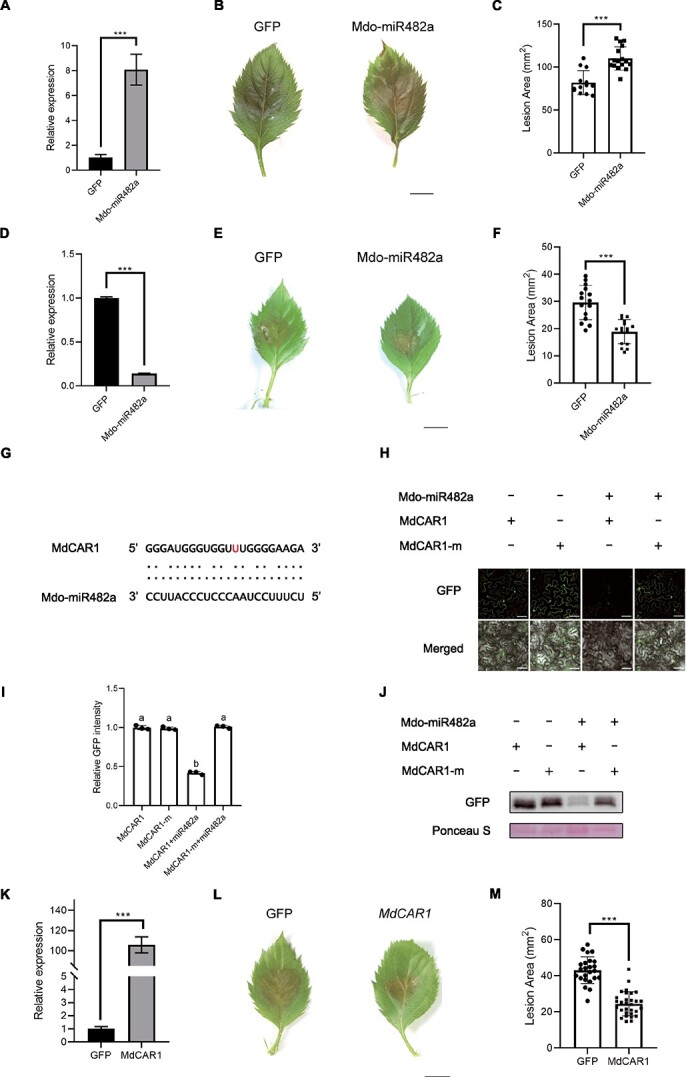
Functional validation of Mdo-miR482a in *Malus domestica*. (**A**) Transcript levels of Mdo-miR482a in Mdo-miR482a-overexpressed plants. Mean ± SD were determined based on three technical replicates. Consistent outcomes were observed across three biological replicates. Relative expression was normalized using the average value of healthy controls. Statistical analysis was conducted using a two-tailed *t*-test, with the wild type as control. ^***^P < 0.001. (**B**, **C**) The overexpression of Mdo-miR482a in apple leaves leads to decreased resistance against *Valsa mali*. Inoculation of *V. mali* wild-type (WT) was performed on apple leaves at 5 d post agro-infiltration. Lesion areas were photographed and measured at 36 h post inoculation. Bar, 5 mm. Each shape marker in the bar graph represents a biological replicate. (**D**) Transcript levels of Mdo-miR482a in Mdo-miR482a-STTM plants. Mean ± SD were determined based on three technical replicates. Consistent outcomes were observed across three biological replicates. Relative expression was normalized using the average value of healthy controls. (**E**, **F**) The silencing of Mdo-miR482a in apple leaves leads to increased resistance against *V. mali*. Inoculation of *V. mali* wild-type (WT) was performed on apple leaves at 5 d post agro-infiltration. Lesion areas were photographed and measured at 24 h post inoculation. Bar, 5 mm. Each shape marker in the bar graph represents a biological replicate. (**G**) The target cleavage sites of *MdCAR1* determined by degradome is showed as red letter. (**H**) The fusion of *GFP* with target sites of *MdCAR1*, along with their mutated versions (*MdCAR1*-m fused *GFP*), was transiently expressed in *Nicotiana benthamiana* leaves, both in the presence and absence of Mdo-miR482a. GFP fluorescence was observed using confocal microscopy. Bar, 100 μm. (**I**) Relative GFP intensity was normalized to the GFP intensity mean of MdCAR1-fused GFP. Error bars represent the SD value of 30 *N. benthamiana* cells. Lowercase letters were used to indicate statistically significant differences (*P*-adjusted <0.05) as determined by performing one-way analysis of ANOVA followed by Tukey’s multiple comparison test. (**J**) Western blotting was employed to detect the presence of fused GFP, with Ponceau S serving as the control. (**K**) Transcript levels of *MdCAR1* in *MdCAR1*-overexpressed plants. Mean ± SD were determined based on three technical replicates. Consistent outcomes were observed across three biological replicates. Relative expression was normalized using the average value of healthy controls. (**L**, **M**) The overexpression of *MdCAR1* in apple leaves leads to increased resistance against *V. mali*. Inoculation of *V. mali* wild-type (WT) was performed on apple leaves at 5 d post agro-infiltration. Lesion areas were photographed and measured at 28 h post inoculation. Bar, 5 mm. Each shape marker in the bar graph represents a biological replicate.

Three apple miRNAs—Mdo-miR482a, Mdo-miR482b, and Mdo-miRN5971—were identified to be specifically up-regulated in the bark, with the potential to target 22 NLR genes. Notably, Mdo-miR482a and Mdo-miR482b collectively target 21 Md-NLRs. To validate the sRNA-Seq data, stem-loop quantitative real-time PCR (stem-loop qRT-PCR) was utilized to evaluate alterations in Mdo-miR482a and Mdo-miR482b expression following *V. mali* infection in bark and leaves, using healthy tissue as a control. The results showed that Mdo-miR482a and Mdo-miR482b were exclusively up-regulated in the bark, with no significant changes observed in the leaves upon *V. mali* infection (Fig. S2, see online supplementary material). To further clarify the functional roles of these two miRNAs, we attempted to construct overexpression vectors for both and try to overexpress them in apple leaves. *Agrobacterium*-mediated transformation was successfully employed to overexpress Mdo-miR482a in apple leaves. The overexpressed leaves demonstrated a substantial upregulation of Mdo-miR482a transcript levels compared to the control (Fig. 2A). Moreover, overexpressing Mdo-miR482a resulted in a notable decrease in the resistance of apple leaves against *V. mali* (Fig. 2B and C). To further verify the function of Mdo-miR482a, a vector using Short Tandem Target Mimic (STTM) was constructed, and when it was transiently expressed in apple leaves, the expression of Mdo-miR482a was silenced (Fig. 2D). Importantly, the silencing of Mdo-miR482a led to a significant increase in resistance of apple to *V. mali* (Fig. 2E and F). Additionally, the results of qRT-PCR demonstrated that the expression of some NLR genes predicted as targets of Mdo-miR482a was indeed decreased in overexpressed leaves, especially MD03G1071300 (Fig. S3, see online supplementary material). Based on BLAST, MD03G1071300 was found to be a homolog of CEL-ACTIVATED RESISTANCE 1 (CAR1) in *A. thaliana* [36], so we named it *MdCAR1*. To further verify whether *MdCAR1* could be regulated by Mdo-miR482a, *MdCAR1* and Mdo-miR482a were performed co-expressed in *Nicotiana benthamiana* leaves. It was found that the green fluorescent protein (GFP) intensity of *MdCAR1*-fused *GFP* was significantly reduced when co-expressed with Mdo-miR482a (Fig. 2H and I), compared with the control (*MdCAR1*-fused *GFP*). Importantly, synonymously mutated version of *MdCAR1* at the target region (*MdCAR1*-m) (Fig. 2G) could not be silenced by Mdo-miR482a. Western blotting analysis corroborated that Mdo-miR482a could silence the expression of *MdCAR1* (Fig. 2J). To further validate the function of *MdCAR1*, we successfully overexpressed *MdCAR1* in apple leaves using *Agrobacterium*-mediated transformation (Fig. 2K). The results showed that the overexpression of *MdCAR1* significantly enhanced the resistance of apple leaves to *V. mali* (Fig. 2L and M). These suggested that Mdo-miR482a, a tissue-specific miRNA, had the potential to weaken apple resistance against *V. mali* by targeting a NLR gene *MdCAR1*.

### Identification of novel milRNAs in *V. mali*

To determine the distinct expression profiles of *V. mali* milRNAs upon pathogen infection of varying host tissues, small RNA sequencing data from IBMd and ILMd were utilized to identify novel milRNAs in *V. mali*, which were subsequently designated as IBVm and ILVm. Additionally, three small RNA libraries were generated from *V. mali in vitro* mycelia (MVm) with three independent biological replicates. Comprehensive information pertaining to the sequencing results can be found in Table S1 (see online supplementary material).

Following the exclusion of non-coding RNA sequences, repetitive sequence, conserved miRNAs, and known milRNAs in *V. mali*, the remaining valid data were employed to uncover novel milRNAs within *V. mali*. This process ultimately led to the identification of 42 novel milRNAs, all exhibiting sequences distinct from previously reported *V. mali* milRNAs [37]. To distinguish between them, these novel milRNAs were designated Vm-milRN1 (Vm-milR_novel1) through Vm-milRN42 (Table S2, see online supplementary material).

Further analysis of the sequence characteristics of novel milRNAs revealed their lengths to be primarily distributed across 20, 21, 22 nt. Moreover, these novel *V. mali* milRNAs displayed a marked preference for uracil at the 5′ end (Fig. S4, see online supplementary material). A self-blast of the precursor sequences for these novel milRNAs confirmed that each novel milRNA in *V. mali* possessed a unique precursor.

### Analysis of fungal differentially expressed milRNAs and potential milRNA targets upon infestation of different tissues of host

**Figure 3:**
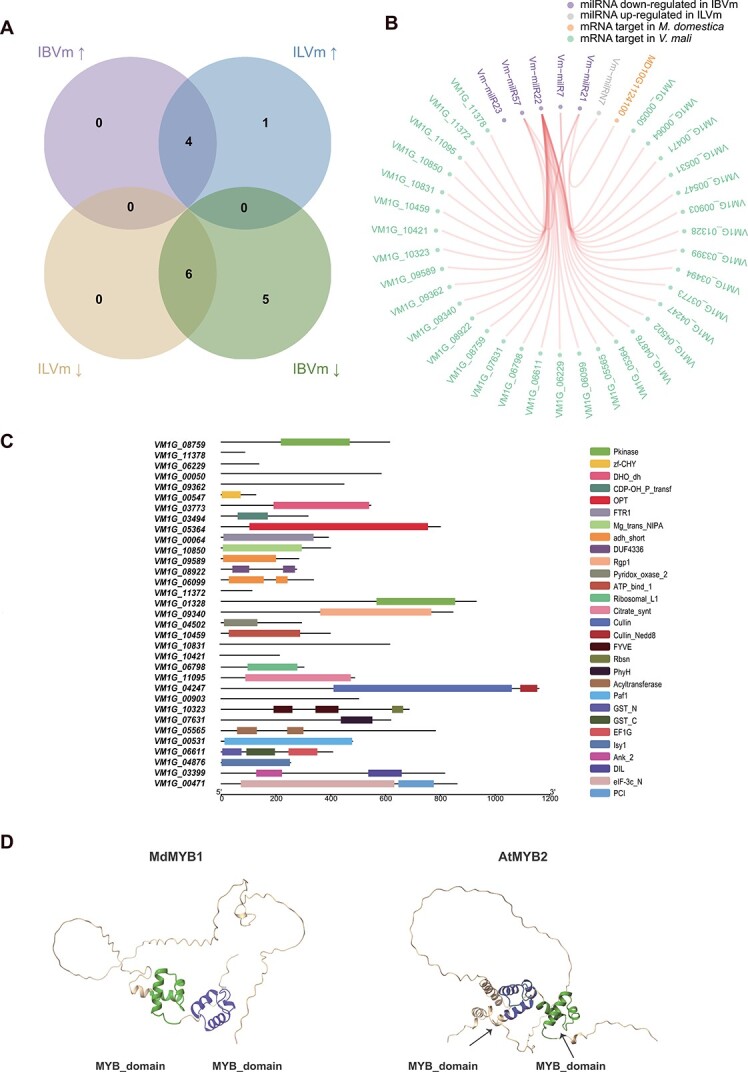
Profile of tsDEMs of *Valsa mali* and the structures of their corresponding target genes when *V. mali* infects different tissues of the host. (**A**) Venn diagram showing the number of up-regulated (up arrows) and down-regulated (down arrows) fungal milRNAs in apple bark and leaves inoculated with *V. mali* (IBVm and ILVm), respectively, at 24 h. (**B**) Network diagram showing the interaction between fungal tsDEMs and their endogenous and exogenous target genes. (**C**) Functional domain structures of endogenous target genes, which were targeted by downregulated tsDEMs in IBVm. (**D**) Tertiary structure of MdMYBT1 and AtMYB2 were predicted by AlphaFold v2. MYB binding domain are marked with the black arrows.

To clarify the transcriptional dynamics of fungal milRNAs infecting various apple tissues, we aligned sequencing data with 99 known and novel *V. mali* milRNAs for quantification purposes (Table S2, see online supplementary material). We then analysed fungal differentially expressed milRNAs (DEMs) in the bark and leaves when infected by the pathogen, using MVm as control. A total of 15 milRNAs (four up-regulated and 11 down-regulated) exhibited significant differential expression in IBVm, while 11 milRNAs (five up-regulated and six down-regulated) were significantly differentially expressed in ILVm. Among these DEMs, five milRNAs (zero up-regulated and five down-regulated) were specifically expressed in IBVm and 1 (one up-regulated and zero down-regulated) milRNA in ILVm (Fig. 3A). We classified these milRNAs as tissue-specific differentially expressed milRNAs (tsDEMs).

To enhance our understanding of the regulatory network involving milRNAs, we performed an analysis to identify and functionally characterize target genes for tsDEMs. Novel milRNA targets were predicted through degradome analysis, while target sequences of known milRNAs were derived from a previously published paper [37]. Moreover, we concentrated solely on endogenous targets (targets in *V. mali*) for down-regulated milRNA expression. Totally five tsDEMs targeted 34 mRNAs in apple and pathogen, with only one milRNA lacking targets. Among these milRNAs, four down-regulated milRNAs had 33 *V. mali* targets, and one up-regulated milRNA in ILVm targeted a single *M. domestica* gene (Fig. 3B).

We performed structural domain predictions and gene functional annotations for all candidate targets to ascertain gene characteristics. Based on these annotations, the functions of 33 genes targeted by down-regulated tsDEMs in IBVm were highly diverse (Fig. 3C; Table S4, see online supplementary material). Notably, these targets were linked to critical functions such as transcriptional regulation (e.g., VM1G_00547), protein transport (e.g., VM1G_09340), energy cycling (e.g., VM1G_05565), and signal transduction (e.g., VM1G_08759). This finding implies that milRNA functions as a negative regulator, promoting pathogen viability and infection by down-regulating during *V. mali* infecting bark tissues. Furthermore, Vm-milRN7, the sole milRNA specifically upregulated in ILVm, was predicted to target an exogenous gene containing two MYB domains (MD10G1124100). A comparison of the tertiary structures of MD10G1124100 and AtMYB2 [38], a reported Arabidopsis gene, revealed significant similarity (Fig. 3D). Consequently, MD10G1124100 likely belongs to the apple MYB transcription factor family.

### Contribution of tissue-specific milRNA Vm-milR57 to pathogenicity and characterization of its candidate targets

Through sRNA-Seq and degradome analysis, we identified four down-regulated tsDEMs targeting endogenous genes in IBVm, demonstrating potential tissue specificity. To further identify tissue-specific expression of milRNA, we compared the expression (transcripts per million, TPM) and fold change of these four milRNAs (Fig. S5, see online supplementary material). Vm-milR57, an milRNA with significantly down-regulated expression in IBVm and negligible changes in expression in ILVm, was selected for further study due to its potential for pronounced tissue specificity. Furthermore, the expression levels of Vm-milR57 in various tissues were corroborated through qRT-PCR, yielding consistent outcomes with the sRNA-Seq analysis. Vm-milR57 was down-regulated when *V. mali* infected the bark, but showed no significant change when infected the leaves (Fig. S6, see online supplementary material). To investigate the contribution of Vm-milR57 to tissue-specific pathogenicity, we generated Vm-milR57 precursor overexpression transformants and Vm-milR57 silencing mutants. Two Vm-milR57 precursor overexpression transformants (OE-3 and OE-4) exhibited a significant upregulation of transcript levels, with a six-fold and five-fold increase, respectively (Fig. S7A, see online supplementary material). The transcript levels of Vm-milR57 in two STTM-silenced mutants (STTM-49 and STTM-102) were significantly down-regulated, with 45%–60% silencing efficiency (Fig. S7B, see online supplementary material).

**Figure 4:**
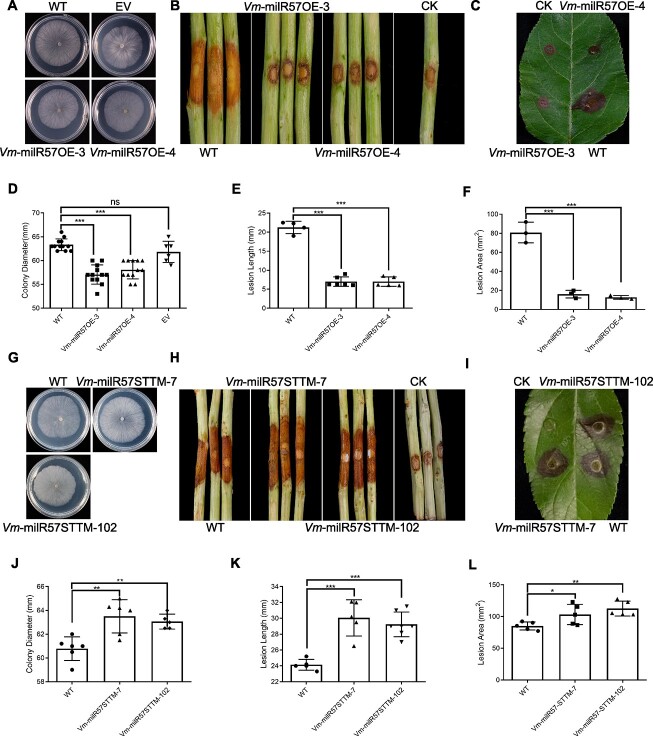
Vegetative growth rate and pathogenicity test of Vm-milR57 precursor overexpression transformants and silence mutants. (**A**, **D**) Vm-milR57 precursor overexpression transformants showed decreased vegetative growth rate. The photograph was taken 3 d after inoculation. (**B**, **E**) Vm-milR57 precursor overexpression transformants showed decreased pathogenicity at the time of inoculation of apple twigs. The photograph was taken 4 d after inoculation. (**C**, **F**) Vm-milR57 precursor overexpression transformants showed decreased pathogenicity at the time of inoculation of apple leaves. The photograph was taken 36 h after inoculation. (**G**, **J**) Vm-milR57 silence mutants show increased vegetative growth rate. The photograph was taken 4 d after inoculation. (**H**, **K**) Vm-milR57 silence mutants show increased pathogenicity at the time of inoculation of apple twigs. The photograph was taken 4 d after inoculation. (**I**, **L**) Vm-milR57 silence mutants show decreased pathogenicity at the time of inoculation of apple leaves. The photograph was taken 36 h after inoculation. *Valsa mali* strain 03–8 was used as wild-type (WT). PDA plugs were used as the control (CK). Each shape marker in the bar graph represents a biological replicate. Statistical analysis was conducted using a two-tailed *t*-test, with the wild type as control. ^*^P < 0.05; ^**^P < 0.01, ^***^P < 0.001; ns, not significant.

Vm-milR57OE-3 and Vm-milR57OE-4 displayed a minor decrease in vegetative growth compared to wild-type (WT) strains (Fig. 4A and D). The pathogenicity of the transformants was significantly reduced on both bark and leaves, by 67% on bark (Fig. 4B and E) and 80–84% on leaves (Fig. 4C and F). These results confirmed that while Vm-milR57 had minimal impact on fungal vegetative growth, it played a crucial role in *V. mali* pathogenicity, particularly when infecting leaves.

STTM-silenced mutants of milR57 exhibited a slight increase in vegetative growth rate (Fig. 4G and J). The silenced mutants demonstrated increased pathogenicity on both bark and leaves compared to WT based on lesion size, with 21–24% increase in pathogenicity on bark (Fig. 4H and K) and 21–32% increase in pathogenicity on leaves (Fig. 4I and L). This indicated that Vm-milR57 silencing suppressed its transcriptional level, enhancing the expression of the target gene and consequently increasing *V. mali* pathogenicity. Similar to overexpression transformants, silencing had a more significant impact on pathogenicity when infecting leaves. These results further validated that milR57 is a negative regulator associated with pathogenesis, with a more notable effect on *V. mali* pathogenicity when infecting leaves.

Previous studies have reported that Vm-milR57 contained four potential targets in *V. mali*. The diminished pathogenicity of Vm-milR57OE and the enhanced pathogenicity of Vm-milR57 silenced mutants imply that certain target genes regulated by Vm-milR57 are likely associated with *V. mali* pathogenicity. To further explore the characteristics of these four target genes, we conducted structural, functional, and phylogenetic analyses. The proteins encoded by these genes included VM1G_00547 as a transcription factor (TF) with a zf-CHY domain, VM1G_10421 as a protein of unknown function, VM1G_06099 as a short-chain dehydrogenase, and VM1G_08759 as a serine/threonine kinase (Fig. 3C). All four targets were predicted to lack signal peptides, suggesting they function intracellularly and are not secreted proteins.

To thoroughly analyse the four targets in the fungal kingdom, we conducted a comprehensive search for orthologs among the genomes of 540 fungal species, encompassing 368 Ascomycota, 121 Basidiomycota, and 51 lower fungi (Fig. S8A, see online supplementary material). By assessing their conservation (Fig. S8B, see online supplementary material) and distribution (Fig. S8C, see online supplementary material) across the fungal kingdom, we ranked the four genes from most conserved to least conserved as VM1G_08759, VM1G_06099, VM1G_00547, and VM1G_10421. VM1G_10421 had few orthologs in *Sordariomycetes*, indicating that it may be a newly evolved gene in *Valsa*. The other three genes were homologous in Ascomycota, Basidiomycota, and lower fungi, demonstrating their higher conservation within the fungal kingdom.

### Validation of Vm-milR57 silencing targets

In order to assess the regulation of four candidate genes by Vm-milR57, we investigated their expression levels in *V. mali* during both infected and uninfected phases. Utilizing the data obtained from qRT-PCR, we identified two target genes, VM1G_06099 and VM1G_00547, which exhibited increased expression trends during *V. mali* infecting apple bark (Fig. S9A, see online supplementary material), but the change in expression was not significant in leaves (Fig. S9B, see online supplementary material). Subsequent gene annotations revealed that VM1G_06099 and VM1G_00547 encoded short-chain dehydrogenase/reductases enzyme and CHY-type zinc finger protein, respectively, and were thus designated as *VmSDR1* and *VmCHY1*.

To confirm the potential suppression of *VmSDR1* and *VmCHY1* by Vm-milR57, we examined their expression levels in wild-type (WT), Vm-milR57 precursor overexpression transformant, and Vm-milR57 silencing mutant strains. Using the WT as a control, we observed significant downregulation of *VmSDR1* and *VmCHY1* expression in the Vm-milR57 overexpression transformant, whereas significant upregulation was noted in the Vm-milR57 silencing mutant (Fig. S10, see online supplementary material), indicating negative regulation of these two genes by Vm-milR57.

**Figure 5:**
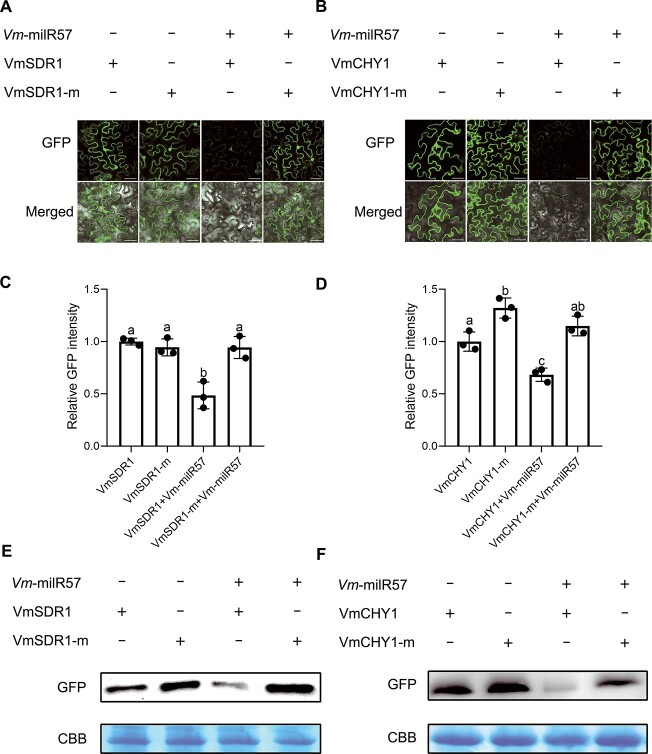
Vm-milR57 silences *VmSDR1* and *VmCHY1* in a sequence-specific manner. (**A**, **B**) The fusion of *GFP* with target sites of *VmSDR1* and *VmCHY1*, along with their mutated versions (*VmSDR1*-m and *VmCHY1*-m fused *GFP*), was transiently expressed in *Nicotiana benthamiana* leaves, both in the presence and absence of Vm-milR57. GFP fluorescence was observed using confocal microscopy. Bar, 100 μm. (**C**, **D**) Relative GFP intensity was normalized to the GFP intensity mean of VmSDR1- or VmCHY1-fused GFP. Error bars represent the SD value of 30 *N. benthamiana* cells. Lowercase letters were used to indicate statistically significant differences (*P*-adjusted <0.05) as determined by performing one-way analysis of ANOVA followed by Tukey’s multiple comparison test. (**E**, **F**) Western blotting was employed to detect the presence of fused GFP, with Coomassie brilliant blue (CBB) serving as the control.

To further substantiate the regulation of *VmSDR1* and *VmCHY1* by Vm-milR57, we performed co-expression experiments in *N. benthamiana* leaves. The results showed that GFP intensity of VmSDR1- and VmCHY1-fused GFP constructs was in a notable decrease when co-expressed with Vm-milR57 (Fig. 5A–C and D), compared to the respective controls. Importantly, synonymous point mutant versions of *VmSDR1* and *VmCHY1* within the target region (*VmSDR1*-m and *VmCHY1*-m) were not silenced by Vm-milR57. The stem-loop RT-PCR results demonstrate that Vm-milR57 is indeed expressed when co-expressed with the target gene in *N. benthamiana* leaves (Fig. S11, see online supplementary material). Additionally, Western blotting analysis corroborated that Vm-milR57 effectively silenced *VmSDR1* and *VmCHY1*, while their respective mutants were unaffected (Fig. 5E and F). The above results indicated that Vm-milRNA57 exerts sequence-specific regulation on the expression of *VmSDR1* and *VmCHY1*.

### Effects of two target genes of Vm-milR57 on pathogenicity of *V. mali*

**Figure 6:**
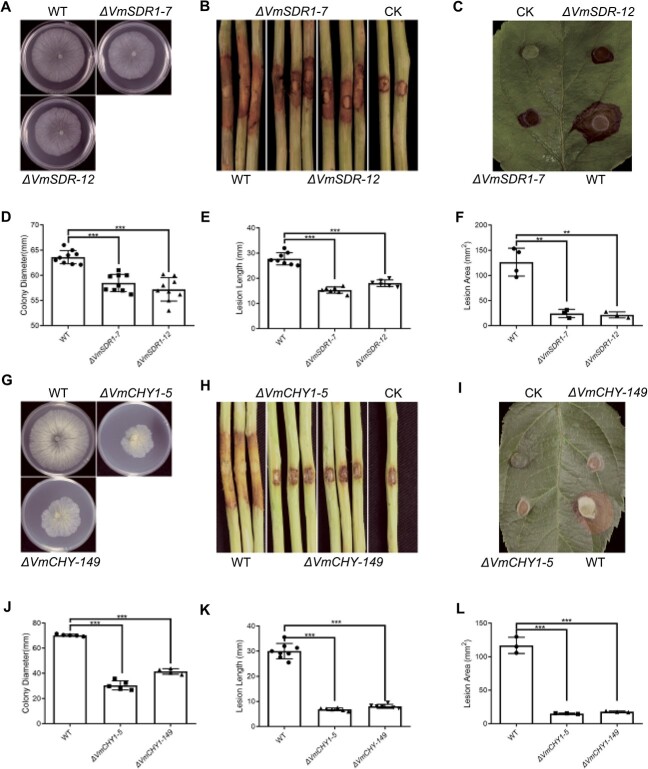
*VmSDR1* and *VmCHY1* are closely associated with pathogenicity of *Valsa mali*. (**A**, **D**) *ΔVmSDR1* mutants showed slightly reduced in vegetative development. The photograph was taken 3 d after inoculation. (**B**, **E**) *ΔVmSDR1* mutants showed reduced in pathogenicity at the time of inoculation of apple twigs. The photograph was taken 4 d after inoculation. (**C**, **F**) *ΔVmSDR1* mutants showed reduced in pathogenicity at the time of inoculation of apple leaves. The photograph was taken 36 h after inoculation. (**G**, **J**) *ΔVmCHY1* mutants showed significantly reduced in vegetative development, and colony morphology was deformed. The photograph was taken 3 d after inoculation. (**H**, **K**) *ΔVmCHY1* mutants almost completely lost pathogenicity at the time of inoculation of apple twigs. The photograph was taken 4 d after inoculation. (**I**, **L**) *ΔVmCHY1* mutants almost completely lost pathogenicity at the time of inoculation of apple leaves. The photograph was taken 36 h after inoculation. *V. mali* strain 03–8 was used as wild-type (WT). PDA plugs were used as the control (CK). Each shape marker in the bar graph represents a biological replicate. Statistical analysis was conducted using a *t*-test, with the wild type as control. ^**^P < 0.01, ^***^P < 0.001.

Overexpressing Vm-milRNA57 mutants led to a reduction in pathogenicity, while the silence mutants exhibited an increase in it. Vm-milRNA57 significantly inhibited the expression of *VmSDR1* and *VmCHY1*, both of which were identified as crucial components contributing to *V. mali* pathogenicity. Homologous recombination was employed to generate deletion mutants of *VmSDR1* and *VmCHY1* (Fig. S12, see online supplementary material), with each target gene possessing. In comparison to the control strain 03–8, both *VmSDR1* and *VmCHY1* deletion mutants exhibited a decrease in vegetative growth, with *ΔVmSDR1* exhibiting a more pronounced growth reduction and colony morphology distortion (Fig. 6A, D, G, and J). Moreover, both *ΔVmSDR1* and *ΔVmCHY1* showed a significant reduction in pathogenicity, with *ΔVmCHY1* displaying almost complete loss of pathogenicity in both inoculated twigs and leaves (Fig. 6H, I, K, and L). Notably, the decrease in pathogenicity of *ΔVmSDR1* demonstrated variance in different tissues, with a 35–45% reduction on bark (Fig. 6B and E) and 81–83% on leaves (Fig. 6C and F). The complementation strains of *ΔVmSDR1* and *ΔVmCHY1* exhibited normal phenotype identical to the wild-type (Fig. S13, see online supplementary material). These results suggested that *VmSDR1* and *VmCHY1* were closely associated with pathogenicity of *V. mali*, and the effect of *VmSDR1* on pathogenicity showed a tissue preference.

## Discussion

The symptoms of plant diseases are often restricted to the specific parts of host plants, suggesting that various pathogens specifically interact with particular host tissues or organs [2]. Different plant tissues exhibit distinct levels of resistance to pathogens, and tissue specificity of plant-pathogen interactions may influence pathogen pathogenicity [2]. For example, maize stems and ears could reduce the pathogenicity of *U. maydis* by high expression of the antifungal protein KP4, whereas leaves seem to lack this resistance mechanism, which causes the pathogen to preferentially infect the leaves [39]. *P. palmivora* develops appressoria to penetrate host cells during colonization of barley roots, but cannot produce appressoria when colonizing leaves, preventing *P. palmivora* from infecting leaves [6]. Although the tissue specificity of plant-pathogen interactions arises from a complex co-evolutionary process, many aspects of its formation mechanism remain undefined. In particular, factors exhibiting distinct regulation patterns during interactions between pathogens and various host tissues require further investigation.

miRNAs/milRNAs genetic regulation represents a key mechanism in plant-pathogen interactions [40]. Most miRNAs/milRNAs can target endogenous genes, contributing to the reprogramming of gene expression to balance plant immunity and pathogen pathogenicity [41]. Recent investigations have unveiled that miRNAs/milRNAs could silence exogenous target genes through cross-kingdom RNAi [18, 31, 42], playing a crucial role in plant-pathogen interactions. Although extensive research demonstrating that tissue specific dynamics are involved in numerous aspects of plant-pathogen interactions, the role of miRNAs in the tissue specificity interactions remains underexplored. To address this gap, the interactions between *V. mali* and apple different tissues were used to analyse the function of miRNAs/milRNAs in tissue-specific interactions regulation.

In this study, sRNA-Seq analysis was conducted to identify tissue-specific differentially expressed miRNAs (tsDEMs) in both pathogen and host by comparing expression levels of miRNAs when *V. mali* interacted with different apple tissues. The number of apple tsDEMs in bark and leaves responding to *V. mali* infection showed considerable differences. When the apple leaves were challenged with *V. mali*, almost no miRNA was differentially expressed, suggesting that miRNA may not play a key regulatory role in apple leaves’ response to pathogen attack. In contrast, miRNAs exhibited an essential role in the response of apple bark to *V. mali* infection. Interestingly, one miRNA, Mdo-miR482a was found to play negative roles in apple resistance to *V. mali*, and it could suppress the expression of a NLR gene *MdCAR1*, which was a candidate resistance related gene of apple. Actually, Mdo-miR482a is a conserved member of miR482/2118 superfamily, which has been identified in many plants [43, 44]. MiR482/2118 predominantly targets NLR genes in eudicots [45, 46]. Because plants conventionally rely on NLR genes to activate immune responses [47], the miR482/2118 family is supposedly a key regulator of disease resistance in eudicots [45]. For example, miR482 in tomato negatively regulates plant resistance to *Phytophthora infestans* [48], which is similar to our results in apple. As Mdo-miR482a is only up-regulated in pathogen-infected bark tissues, it is speculated that Mdo-miR482a facilitates *V. mali* infecting apple bark by suppressing the resistance mediated by *MdCAR1*. Although the module of Mdo-miR482 and *MdCAR1* is likely to be a conserved mechanism in eudicots, it is also very interesting that the module could be specifically activated in the specific interaction system between *V. mali* and bark tissues. However, how the expression of Mdo-miR482a in bark tissue is specifically induced by *V. mali* is worthy of further research.

Not only do apple miRNAs exhibit tissue specificity in response to *V. mali* infection, but milRNAs of *V. mali* also share a similar characteristic. Previous studies have shown that milRNAs in *V. mali* can target the apple gene cross-kingdom such as RLKs [31]. Here, we identified a Vm-milRNA that was up-regulated in leaves during the infectious stage, potentially targeting a host MYB transcription factor. Plants’ MYB transcription factors play a critical role in regulating the synthesis of phenylpropanoid-derived compounds, which contributes to plant growth, development, and defense against biotic and abiotic stress [49]. *V. mali* might deliver milRNAs to apple leaf cells to influence the production of phenylpropanoid-derived compounds, thereby reducing the host’s defense capabilities. It should be noted that this speculation is only based on sequence features and degradation analysis, and further research is needed to confirm it. Meanwhile, some specially down-regulated tsDEMs of *V. mali* were also identified when it infected apple bark tissues. Vm-milR57 was a classic example, which was confirmed to be a negative regulator of pathogenesis by inhibiting the expression of *VmSDR1* and *VmCHY1* during *V. mali* infecting apple bark. *VmSDR1* and *VmCHY1* possess conserved short-chain dehydrogenase/reductases and CHY-type zinc finger domains, respectively, and the functions of their homologous in filamentous pathogens have been reported. For example, *FgChy1*, the homolog of *VmCHY1* in *Fusarium graminearum*, is required for pathogenicity. Deletion of *FgCHY1* results in the inability of *F. graminearum* to form infection structure and penetrate through the host epidermis [50]. *MoSCAD2* is a gene from SDR family in *Magnaporthe oryzae*. Deletion of *MoSCAD2* results in drastic reduction in conidiation and delayed conidia germination [51]. Actually, the pathogenic mode in which *V. mali* increase the expression level of pathogenic factors by decreasing the expression of milRNAs to promote the infection has been reported [37]. However, it is the first time that milRNAs specifically participate in the regulation of pathogen infection to different tissues. Based on the above studies, we propose that *V. mali* possesses adaptive regulatory mechanisms mediated by milRNAs when infecting distinct tissues. Vm-milR57 is a tissue-specific regulator that can only be down-regulated and functions when *V. mali* infects bark tissues. It may serve as an important point for studying this regulatory mechanism.

**Figure 7:**
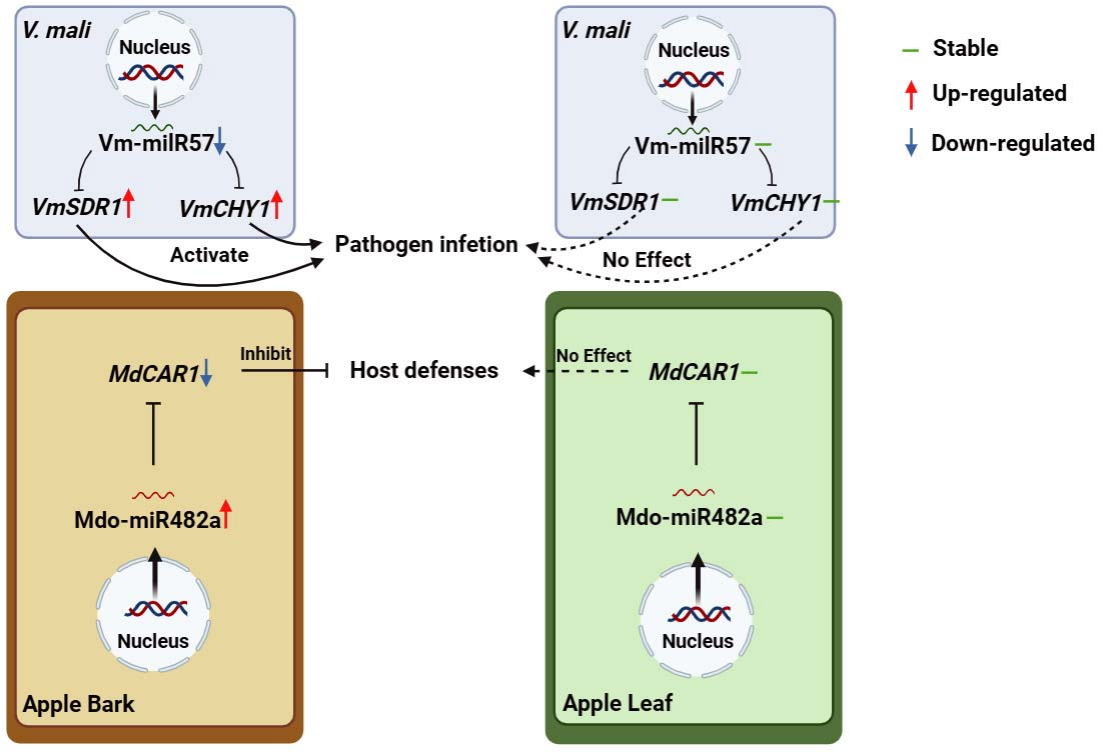
Proposed model for the adaptive regulation of two key miRNAs/milRNAs in tissue-specific interaction between apple and *Valsa mali*. Vm-milR57 is a milRNA downregulated only during *V. mali* infecting apple bark tissues. Its downregulation leads to the upregulation of two pathogenicity-related genes, *VmSDR1* and *VmCHY*1, ultimately enhancing the pathogenicity of *V. mali*. Mdo-miR482a is a miRNA upregulated in apples bark tissues in response to *V. mali* infection. It targets *MdCAR1*, which positively regulates plant resistance, belonging to the NLR gene family. The upregulated expression of Mdo-miR482a leads to the downregulation of *MdCAR1*, thereby reducing the resistance of apple bark to *V. mali*.

In summary, this study represents the first comprehensive analysis of the regulatory network of tissue-specific miRNAs/milRNAs during *V. mali* interacting with apple, expanding the tissue-specific miRNAs/milRNAs library in pathogen-host interactions. Furthermore, two key tissue-specific miRNAs/milRNAs were determined and their functions were explored in the pathogen-infected host, providing new insights of sRNAs’ role in tissue-specific regulation (Fig. 7). Our findings indicate that both the pathogen milRNAs and the host miRNAs are involved in the regulation of tissue-specific interactions between the pathogen and different host tissues, and the regulation mediated by miRNAs/milRNAs contributes to the pathogenesis of tissue preference to a certain extent. It deepens our understanding of the function of miRNAs/milRNAs in the interactions between plants and pathogenic fungi.

## Materials and methods

### Plant and fungal material

Apple (*Malus* × *domestica* Borkh. cv. ‘Fuji’) bark and leaf samples were collected from plants in the experimental field under standard management. The *V. mali* wild-type strain 03–8 were cultured on PDA medium at 25°C.

To generate target gene deletion mutants, we followed previously described methods [37].

### sRNA sequencing and data pre-processing

All sRNA libraries construction and sequencing were performed by Lc-Bio Technologies (Hangzhou, Zhejiang, China). Small RNA sequencing libraries were generated using the TruSeq Small RNA Sample Prep Kits (Illumina, San Diego, CA, USA). The constructed libraries were subjected to sequencing on an Illumina Hiseq2000/2500 platform, employing single-end 1 × 50 bp.

Adaptors, reads outside of 17 nt–25 nt, and low-quality reads were removed by cutadapt v4.1 and Trimmomatic v0.39. rRNA, tRNA, snRNA, snoRNA, and other non-coding RNA sequences were screened out from data using Rfam (https://rfam.xfam.org/), and the source of the repetitive sequences was analysed and filtered with Repbase (http://www.girinst.org/repbase). Then, conserved miRNAs with miRBase (https://www.mirbase.org/) and known Md-miRNAs/Vm-milRNAs were filtered. The filtered data was considered to be the clean data.

### Identification of novel miRNA and DEMs analysis

The clean data were mapped onto known miRNAs of plant and milRNAs of fungi with Bowtie alignment tool v1.3.1. The unaligned reads were mapped to the *M. domestica* genome [52] and *V. mali* genome [53]. Mirdeep2 (https://github.com/rajewsky-lab/mirdeep2) and miR-PREFeR (https://github.com/hangelwen/miR-PREFeR) were employed for the prediction of novel miRNAs and milRNAs. The data containing known miRNAs/milRNAs were aligned against all miRNAs/milRNAs and quantifier module of mirdeep2 was employed for the quantification of expression of all miRNAs/milRNAs.

R (v4.2.1) based DESeq2 package was used to determine the DEMs in each sample. A miRNA was considered to be significantly differentially expressed between the two samples if it exhibited a log2 fold change ≥ | 1 | and FDR ≤ 0.05.

### Degradome sequencing

Two degradome libraries were constructed from samples of the junction of healthy and diseased apple bark or leaves inoculated with *V. mali* at 24 hpi by Lc-Bio Technologies. The prepared library was sequenced by Illumina Hiseq2000/2500, generating single-end reads with a read length of 1 × 50 bp.

### Target prediction and functional analysis

psRNATarget [54] was performed to predict targets. Schema V2 (2017 release) were used to obtain high quality prediction results by setting the maximum expectation to 2. Potentially cleaved targets were performed to identify by CleaveLand v4.3 from degradome-Seq. *M. domestica* mRNA and *V. mali* mRNA were references, respectively. Only targets with a *P*-value <0.05 and category <4 in each sample were identified for discussion.

### Gene structure prediction

The prediction of gene domain was performed by pfam (https://pfam.xfam.org/). The screening threshold was a value of <1.0e-5. Visualization of gene structure was done by TBtools II [55].

Tertiary structures of proteins were predicted by AlphaFold v2 [56] and visualized using the UCSF Chimera tool.

### Identification of fungal orthologs and phylogenetic analysis

Blast+ v2.12.0 [57] was used to find homologs of target genes from data of Ensembl Fungi (https://fungi.ensembl.org/). Bidirectional blastp searches were conducted to identify orthologs. Muscle v3.8.15 [58] was used to generate the sequence alignments and trimAl v1.4.rev15 was used to refine alignments [59]. IQ-tree 2 was used to infer phylogenetic relationships with the ML (maximum likelihood) method [60]. The trees were visualized by R (v4.2.1) based ggtree package [61].

### Relative expression of miRNAs and their corresponding target genes

We extracted total RNA using the miRcute Plant miRNA Isolation Kit (Tiangen, Beijing, China). Expression of miRNA was detected by stem–loop qRT-PCR as described [62]. First-strand cDNA was synthesised by miRNA First Strand cDNA Synthesis (Vazyme, Nanjing, China) with the stem-loop RT primer. The small nuclear RNA U6 (in fungi) and 5.8 rRNA (in plant) was used as control. Transcript levels of genes were analysed by qRT-PCR, which was performed using RealStar Green Power Mixture (GenStar, Beijing, China). MdEF1α of *M. domestica* and G6PDH of *V. mali* were used as the reference genes, respectively. Relative expression of genes was calculated using the 2^-ΔΔCt^ method [63]. All primers used in this study are listed in Table S5 (see online supplementary material).

### Overexpression transformants and silence mutants generation of Vm-milRNA

Vm-milRNA precursors were cloned from *V. mali* genomic DNA and fused with pDL2 using the ClonExpress II single-step cloning kit (Vazyme) and expressed under the control of the *M. grisea* ribosomal protein 27 promoter [64]. Vm-milRNA STTM fragment sequences were designed [65], synthesized, and constructed onto the pDL2-mexp vector by Sangon Biotech (Shanghai, China). Constructs were transformed into *V. mali* wild-type strain 03–8 using the previously established transformation method [66].

### Vegetative growth and pathogenicity tests of *V. mali*

Vegetative growth of *V. mali* strains was assayed as described previously [67]. Briefly, mycelium plugs (d = 5 mm) from the edge of growing colonies were inoculated onto PDA. Pathogenicity was assessed on *M. domestica* Borkh. cv ‘Fuji’ apple twigs and leaves through stab inoculation, following the previously described method [68].

### Plasmid construction

For transient expression of apple-miRNA in *M. domestica*, we cloned Mdo-miR482a and *MdCAR1* from the cDNA of *M. domestica* Borkh. cv. ‘Fuji’ and fused them to pRS300 and pCAMBIA1302 by Tsingke Biotechnology (Beijing, China), respectively. To assess the relationship between Vm-milR57 and targets, we cloned the precursor of Vm-milR57 from *V. mali* genomic DNA and fused it with pCAMBIA1302-GFP using the ClonExpress II single-step cloning kit (Vazyme).

### *Agrobacterium*-mediated transient expression in *N. benthamiana* and apple leaves

An *Agrobacterium*-mediated transient expression assay was performed as previously described [69]. *N. benthamiana* was injected with the *Agrobacterium* suspension and the injection area was marked. After 12 h of dark culture, it was placed in a greenhouse with 16 h light/8 h dark photoperiod for culture. *Malus* × *domestica* ‘GL-3’ [70] apple plantlets were inoculated with Agrobacterium suspension using a vacuum pressure of 65 kPa for a duration of 10 min.

### Confocal microscopy observation

*N. benthamiana* leaves were examined 48 hours after agro-infiltration using an FV3000. GFP fluorescence was excited with a 488 nm wavelength laser, and emission within the range of 505 to 530 nm was detected. GFP intensity calculation was subsequently conducted using IMAGEJ.

### Western blotting

Proteins were extracted using a kit from BestBio (Shanghai, China) and their concentrations were measured with a Tiangen kit. The membranes were incubated with mouse monoclonal antibodies against GFP or actin (from Sungene Biotech, Tianjin, China), and then with a secondary antibody conjugated to HRP from a Beijing-based company.

## Supplementary Material

Web_Material_uhae094
