# Supplementary material for: Adaptive regulation of miRNAs/milRNAs in tissue-specific interaction between apple and Valsa mali
Source: Hortic Res. 2024 Apr 2;11(5):uhae094. doi: 10.1093/hr/uhae094 (PMC11116833; doi:10.1093/hr/uhae094)
Supplement: Web_Material_uhae094 [file web_material_uhae094.zip › supplementary figure revise3.docx]

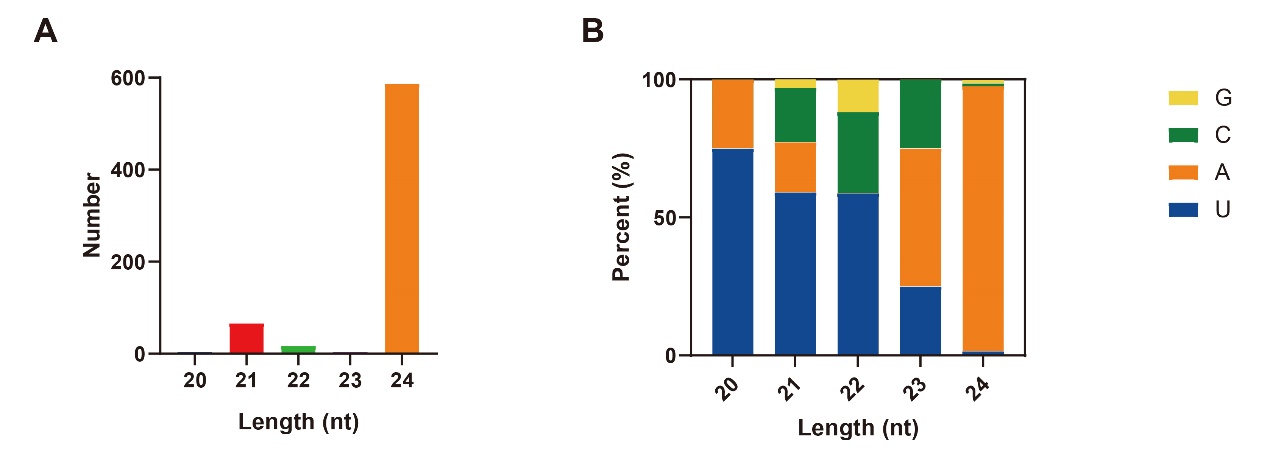


Figure S1. Characteristics of novel miRNAs in *M. domestica*. (A) Length distribution of novel miRNAs of *M. domestica*. (B) First-nucleotide bias of novel miRNAs of *M. domestica*.


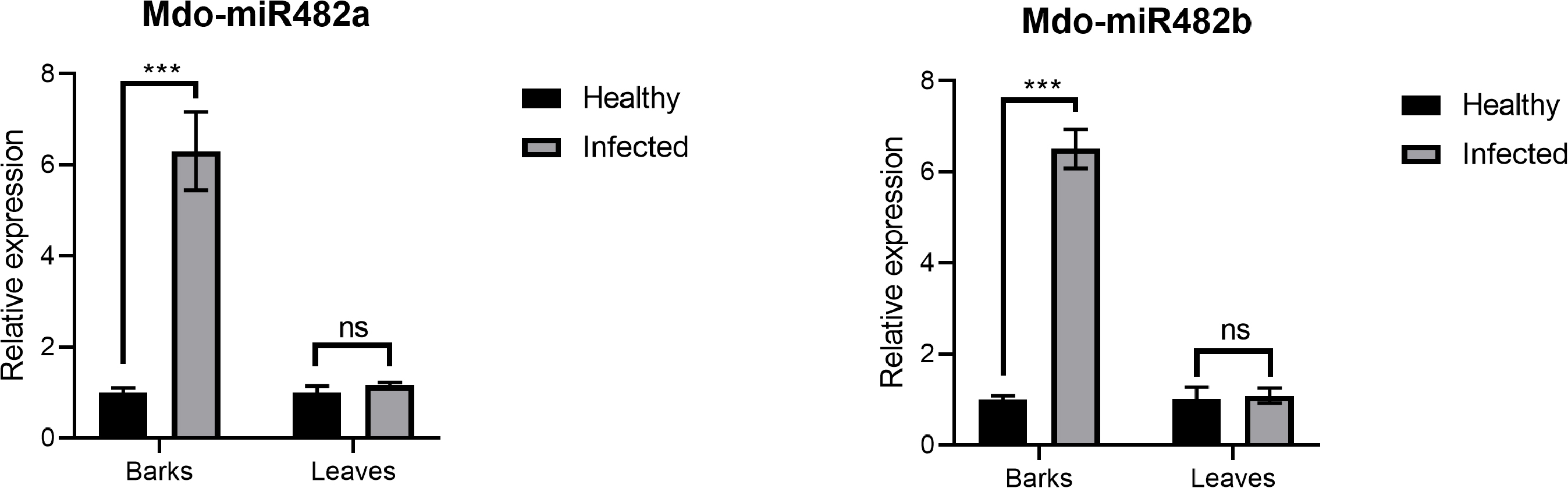


Figure S2. Transcript levels of Mdo-miR482a and Mdo-miR482b in bark and leaves at 24 h post inoculation. Mean ± SD were determined based on three technical replicates. Consistent outcomes were observed across three biological replicates. Relative expression was normalized using the average value of healthy controls. Statistical analysis was conducted using t-test, with the wild type as control. ***, *P* < 0.001; ns, not significant.


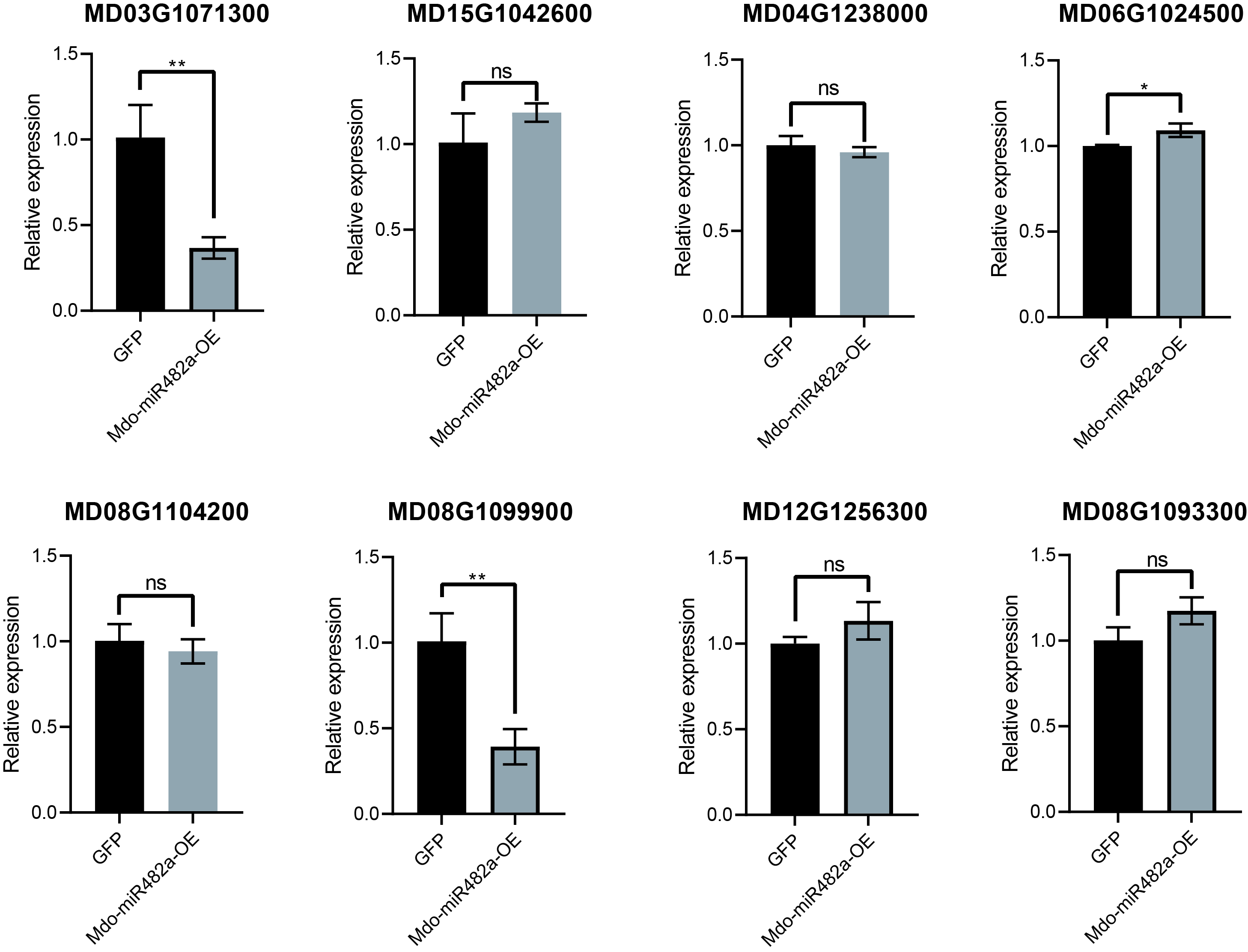


Figure S3. The relative expression levels of all NLR genes in miR482a-overexpressing. Mean ± SD were determined based on three technical replicates. Consistent outcomes were observed across three biological replicates. Relative expression was normalized using the average value of GFP controls. Statistical analysis was conducted using a two-tailed t-test. **, *P* < 0.01. *, *P* < 0.05. ns, no significant.


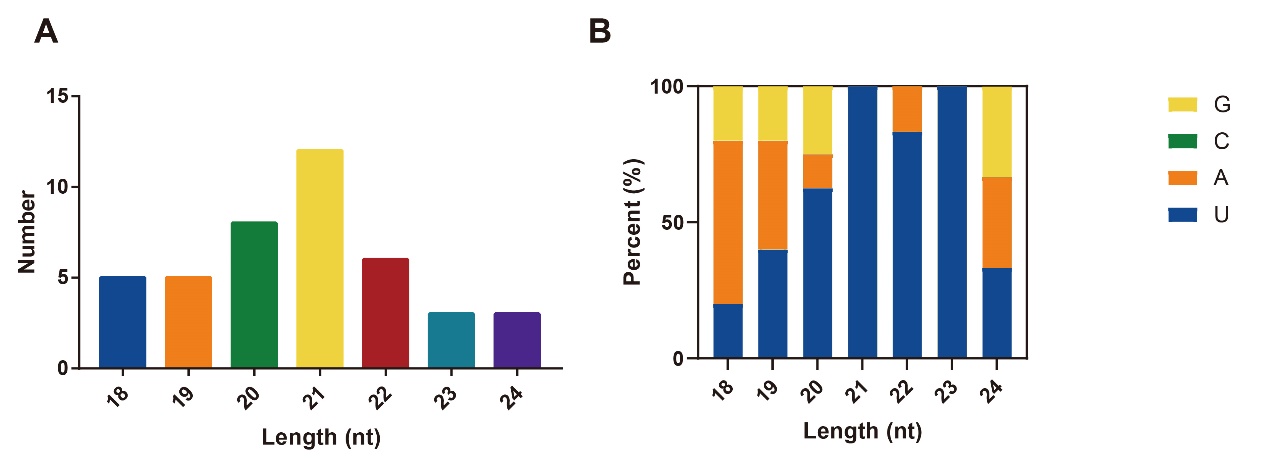


Figure S4. Characteristics of novel milRNAs in *V. mali*. (A) Length distribution of novel milRNAs of *V. mali*. (B) First-nucleotide bias of novel milRNAs of *V. mali*.


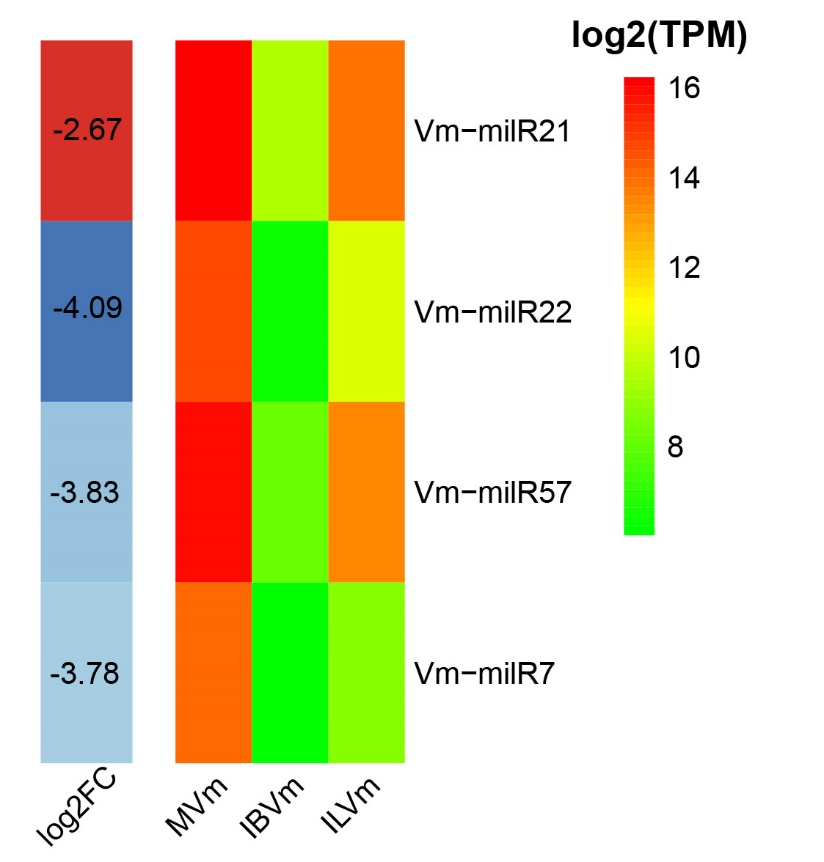


Figure S5. Heat map of the expression patterns of the four downregulated tsDEMs in IBVm.


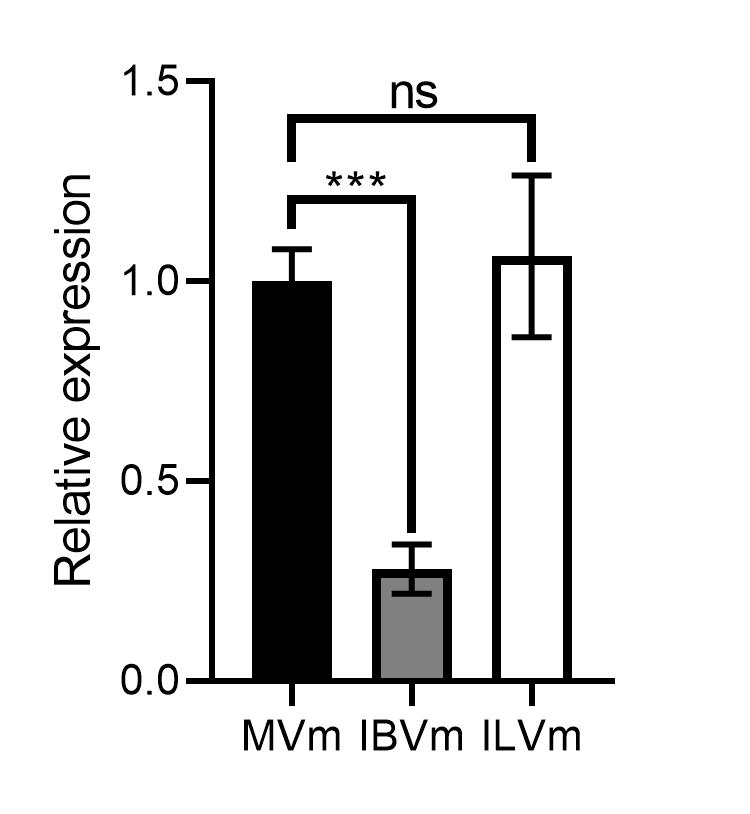


Figure S6. Vm-milR57 is highly induced during *V. mali* bark infection. Mean ± standard deviation (SD) values were calculated from three technical replicates. Similar results were obtained from three biological replicates. MVm represents *V. mali* in vitro cultured mycelia. IBVm represents *V. mali*–apple bark interaction sample at 24 h post-inoculation (hpi). ILVm represents *V. mali*–apple leaves interaction sample at 24 h post-inoculation (hpi). Statistical analyses were performed with a two-tailed t-test by comparing with data of MVm. ***, P < 0.001; ns, not significant.


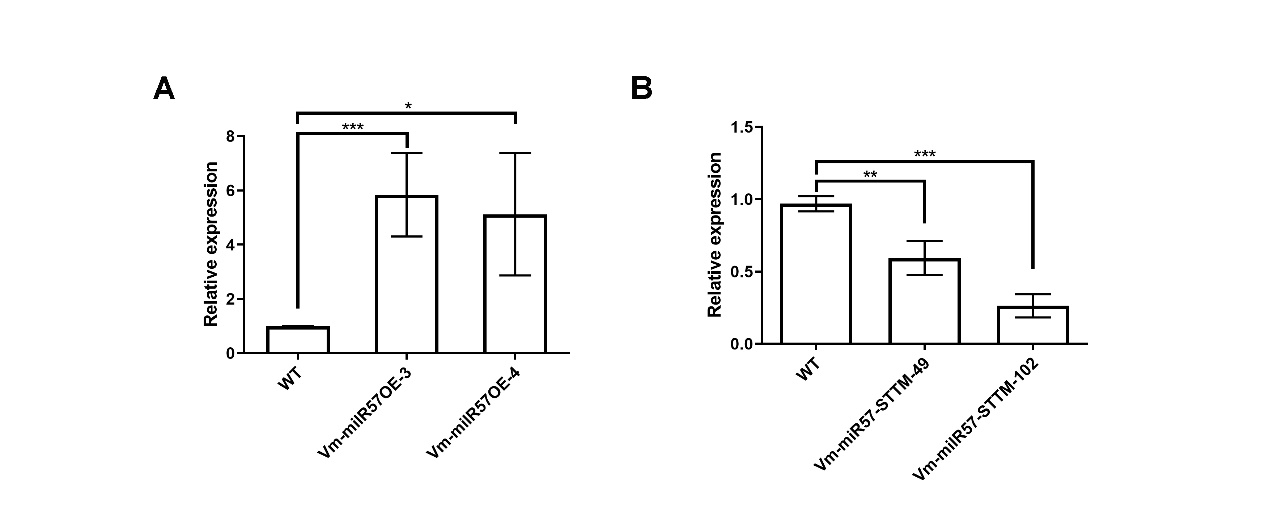


Figure S7. (A) Vm-milR57 show significantly higher relative transcription level in Vm-milR57 overexpression transformants. (B) Vm-milR57 show significantly lower relative transcription level in Vm-milR57 silence mutants. Statistical analyses were performed with two-tailed t-test by comparing with data of wild type. *, P<0.05; **, P<0.01, ***, P<0.001.


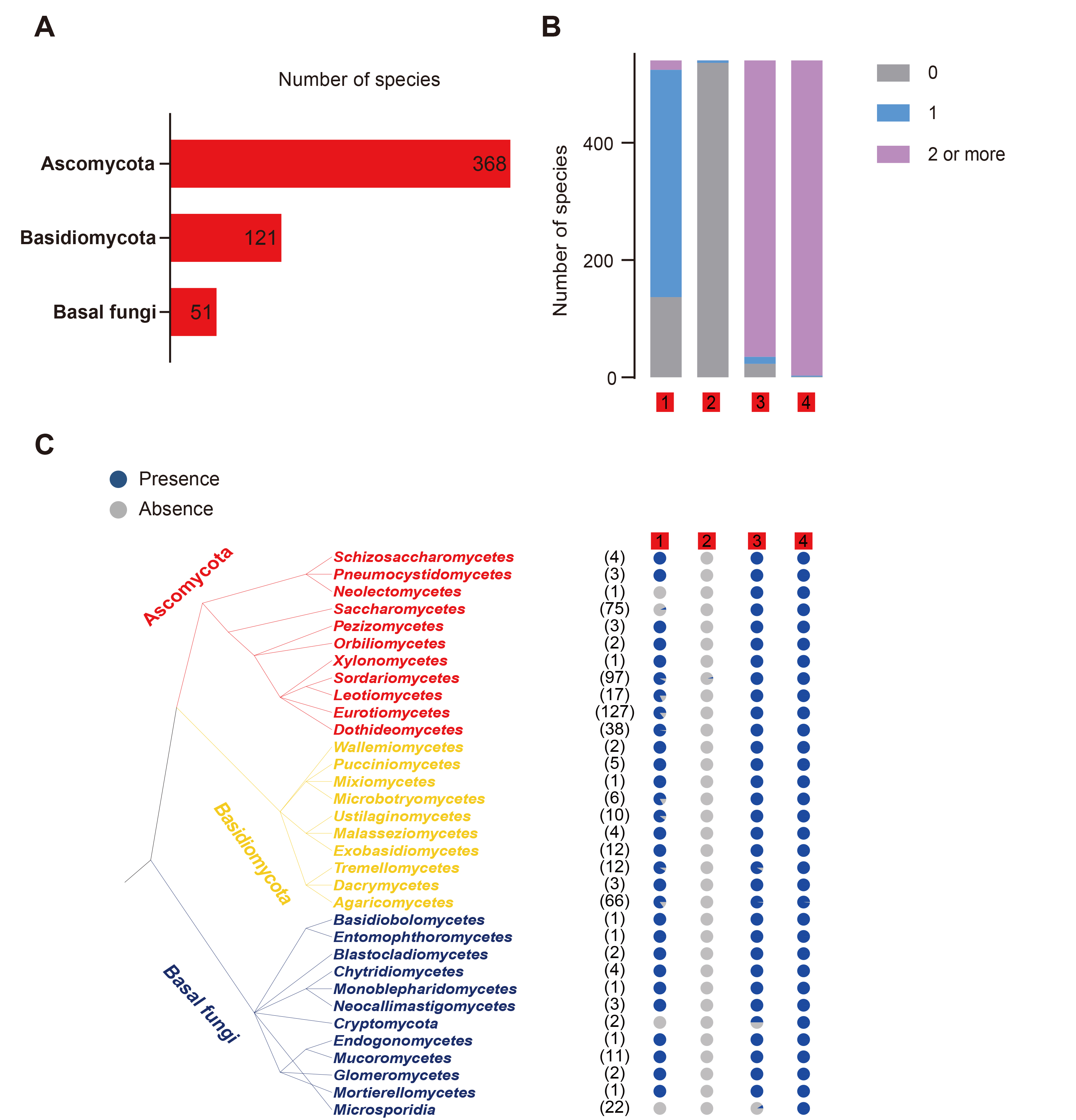


Figure S8. Four target genes of Vm-milR57 in different classes of fungi. (A) The number of fungi belonging to different fungal phyla surveyed in this study. (B) The number of fungal species containing 0 (grey), 1 (blue) or at least 2 copies (pink) of individual target genes (VM1G_00547, VM1G_10421, VM1G_06099, VM1G_08759 labelled as 1, 2, 3, 4 at the bottom). (C) The conservation of four target genes in different lineages across the kingdom Fungi. The dendrogram on the left shows the phylogenetic relationship of the 540 fungi belonging to labeled fungal classes or phyla. The numbers of species in each class or phylum are indicated in the bracket. Individual pie charts in the matrix show the ratio of fungal species in each fungal group with (blue) versus without (grey) individual target genes.


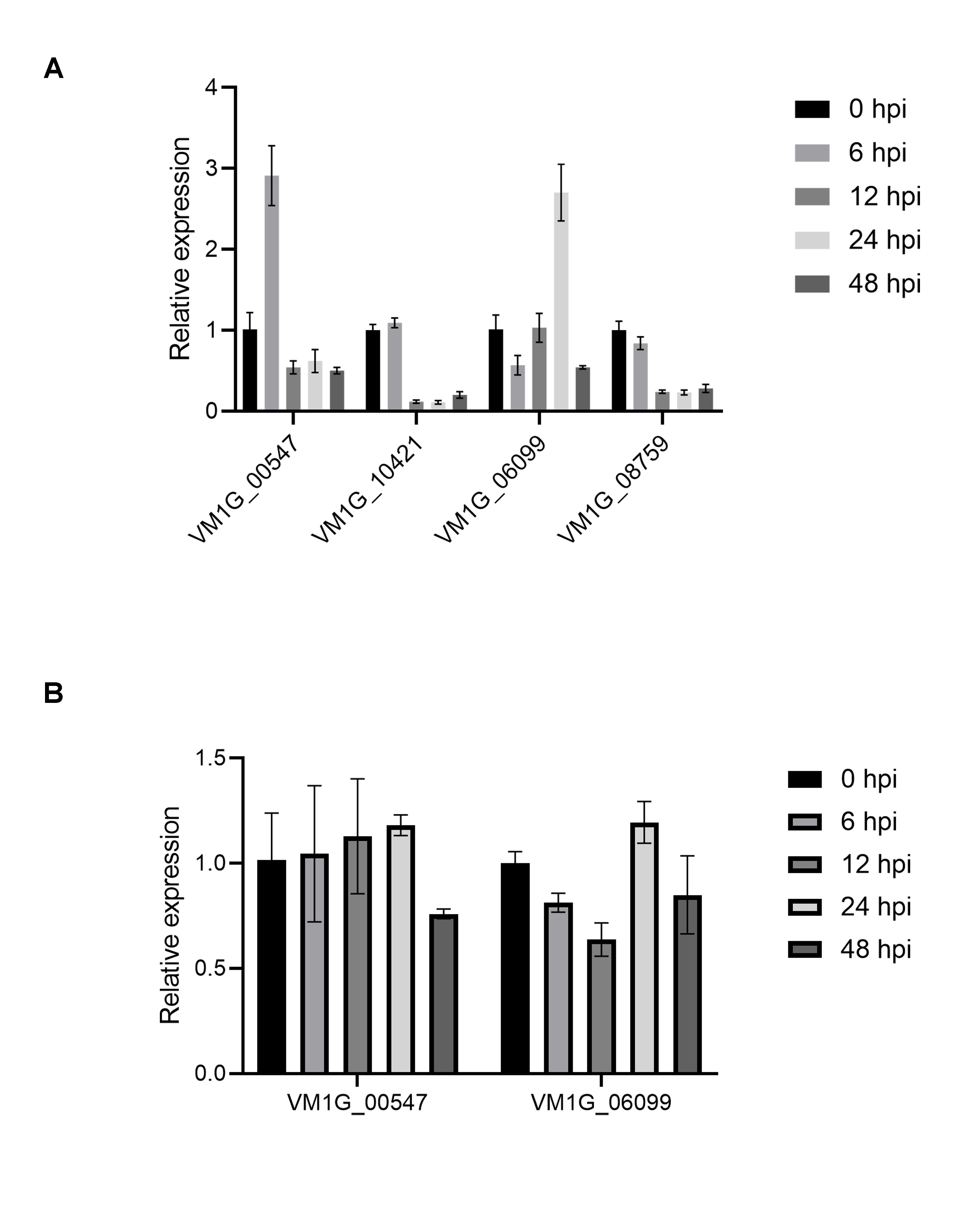


Figure S9. Expression levels of four candidate target genes of Vm-milR57 at different stages of bark infection (A) and leaves infection (B).


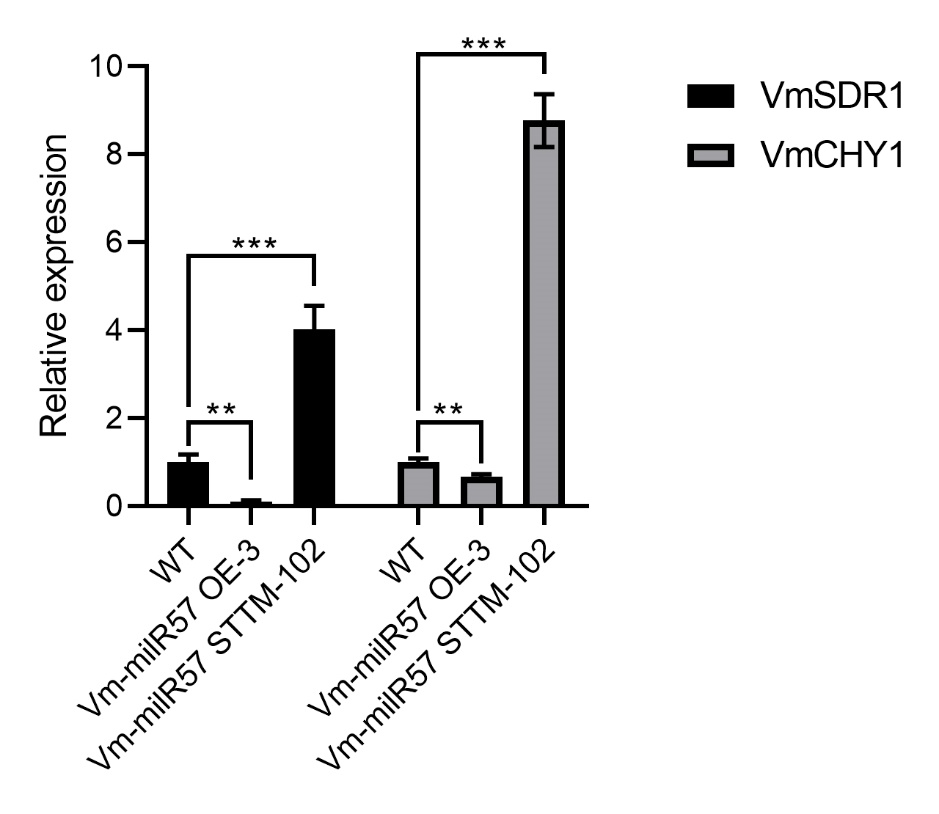


Figure S10. VmSDR1 and VmCHY1 show lower expression level in Vm-milR57 overexpression mutants, while show higher expression level in milR57 silence mutant. The expression of two genes in WT is the control. Statistical analyses were performed with two-tailed t-test by comparing with data of wild type. **, P<0.01, ***, P<0.001.


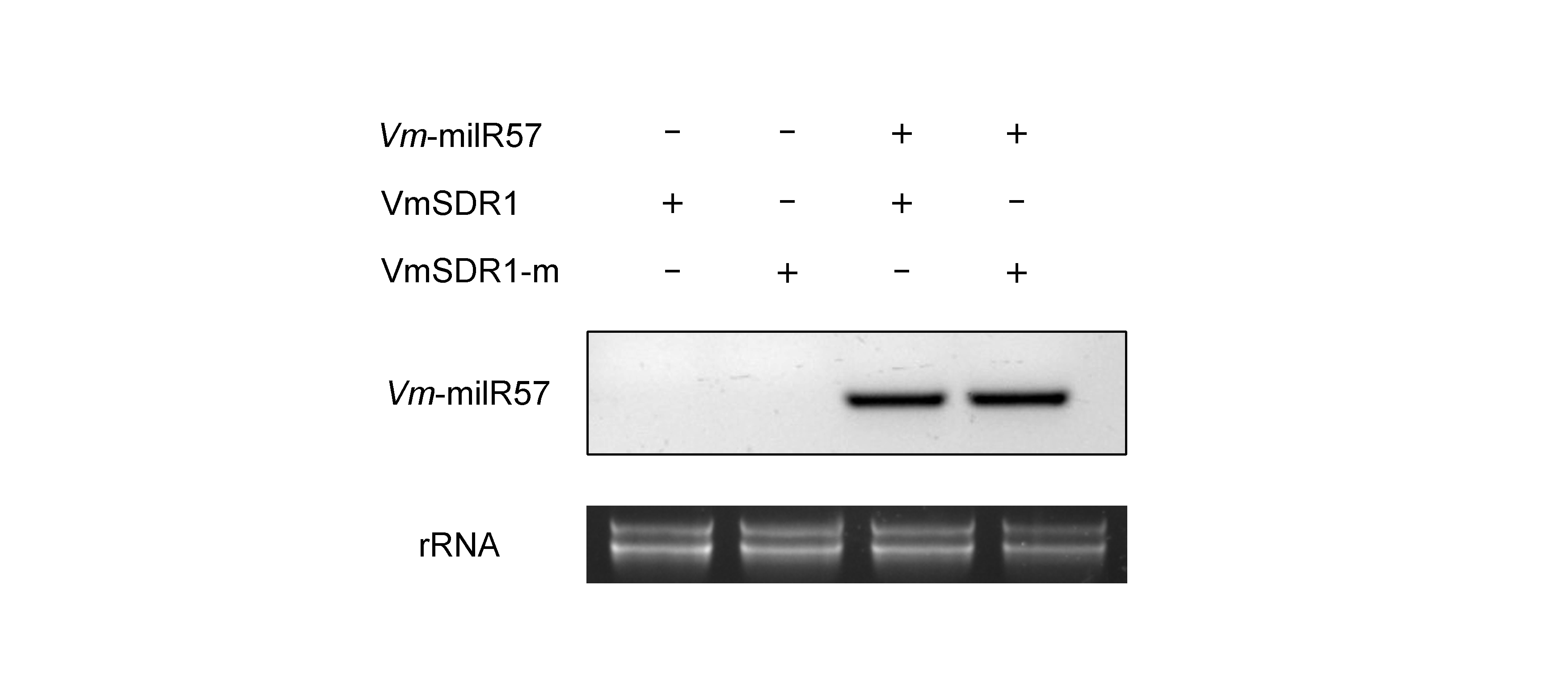


Figure S11. The stem-loop RT-PCR results demonstrate that Vm-milR57 is indeed expressed when co-expressed with the target gene in *N. benthamiana* leaves. rRNA was used as the inner reference control. Similar results were obtained in three biological replicates.


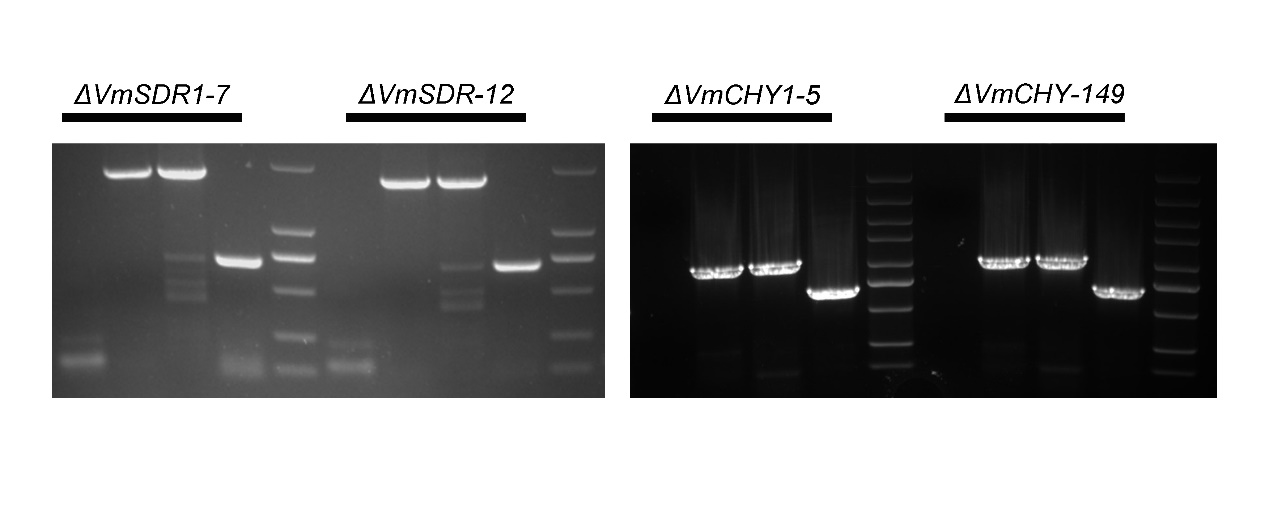


Figure S12. PCR detection of deletion mutants. Four PCR amplifications were utilized to confirm gene deletion in mutants. 1^st^ lanes show gene specific primers 5F/6R designed at the inner part of the target gene were used to verify the deletion of the target gene. 2^nd^ and 3^rd^ two pairs of combined primers gene specific 7F/G855-R and G856-F/gene specific 8R were used to ascertain the targeted homologous recombination upstream or downstream. 4^th^ lanes: primer pair G852-F/G850-R designed at the inner core of G418 resistance coding gene was used to verify the insertion of NEO.


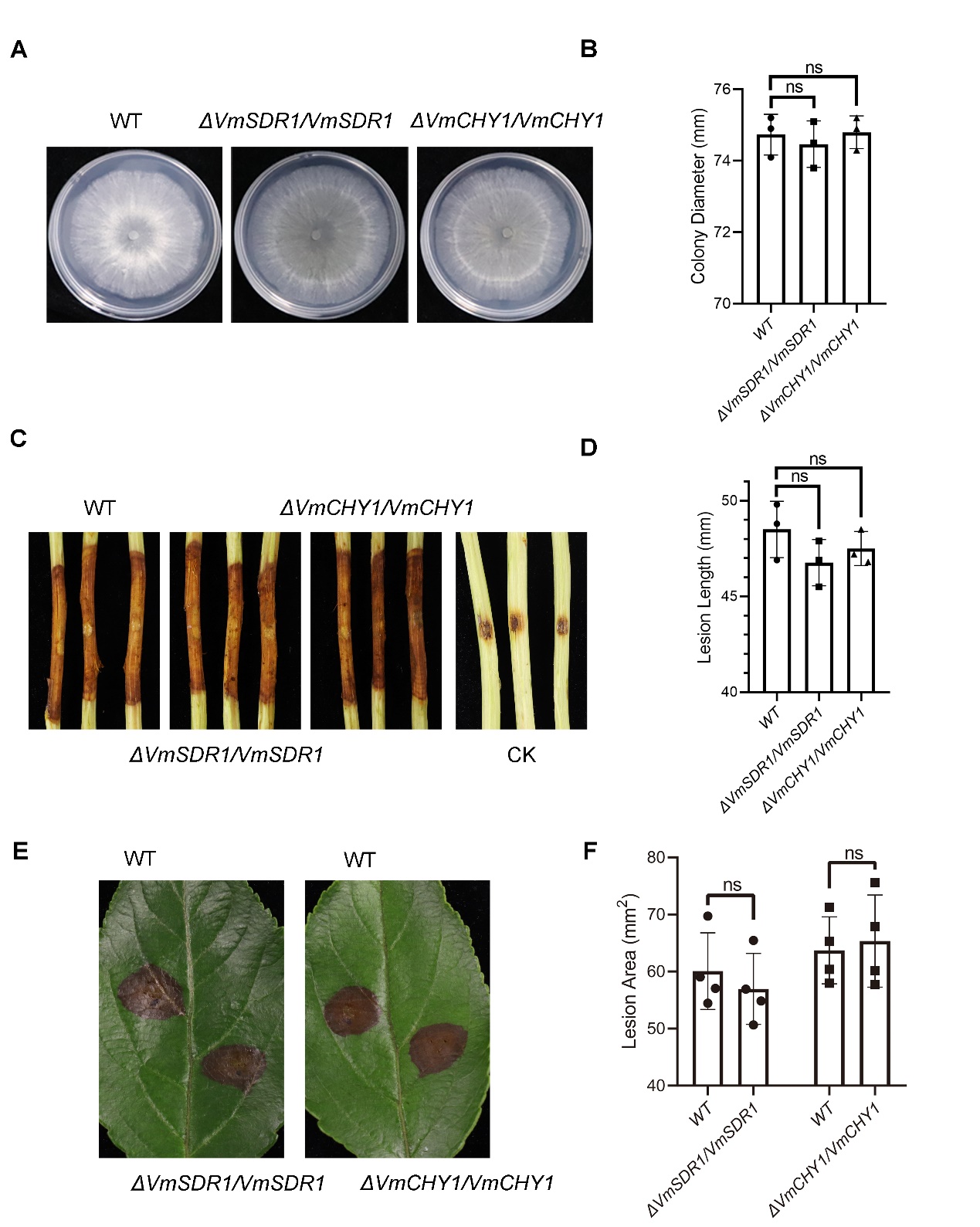


Figure S13. The complementation strains of *ΔVmSDR1* and *ΔVmCHY1* exhibited normal phenotype identical to the wild-type. (A, B) *ΔVmSDR1/VmSDR1* and *ΔVmCHY1/ VmCHY1* strains showed normal phenotype in vegetative development identical to the wild-type. Colony diameters were photographed and measured at 3 d post *V. mali* inoculation. (C, D) *ΔVmSDR1/VmSDR1* and *ΔVmCHY1/ VmCHY1* strains showed normal pathogenicity identical to the wild-type at the time of inoculation of apple twigs. Lesion lengths were photographed and measured at 4 d post *V. mali* inoculation. (E, F) *ΔVmSDR1/VmSDR1* and *ΔVmCHY1/ VmCHY1* strains showed normal pathogenicity identical to the wild-type at the time of inoculation of apple leaves. Lesion areas were photographed and measured at 36 h post *V. mali* inoculation. Each shape marker in the bar graph represents a biological replicate. Statistical analyses were performed with a two-tailed t-test by comparing with data of wild type. ns, not significant.
