# Supplementary figures and images for: Adaptive regulation of miRNAs/milRNAs in tissue-specific interaction between apple and Valsa mali
Source: Hortic Res. 2024 Apr 2;11(5):uhae094. doi: 10.1093/hr/uhae094 (PMC11116833; doi:10.1093/hr/uhae094)

**A**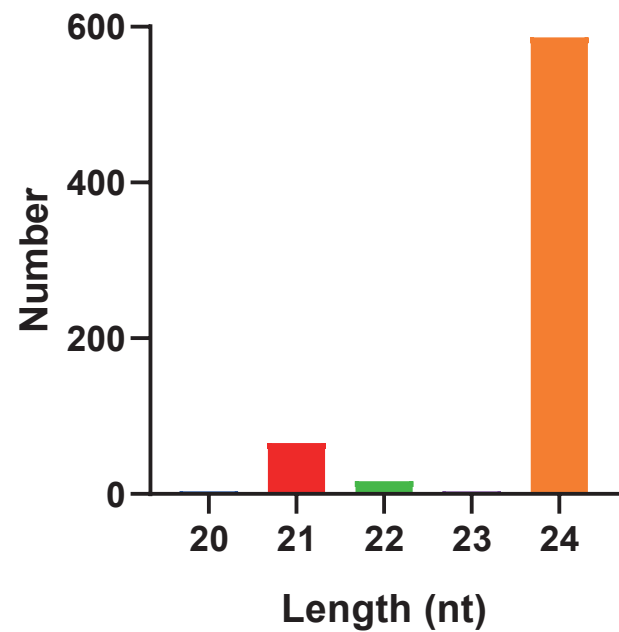**B**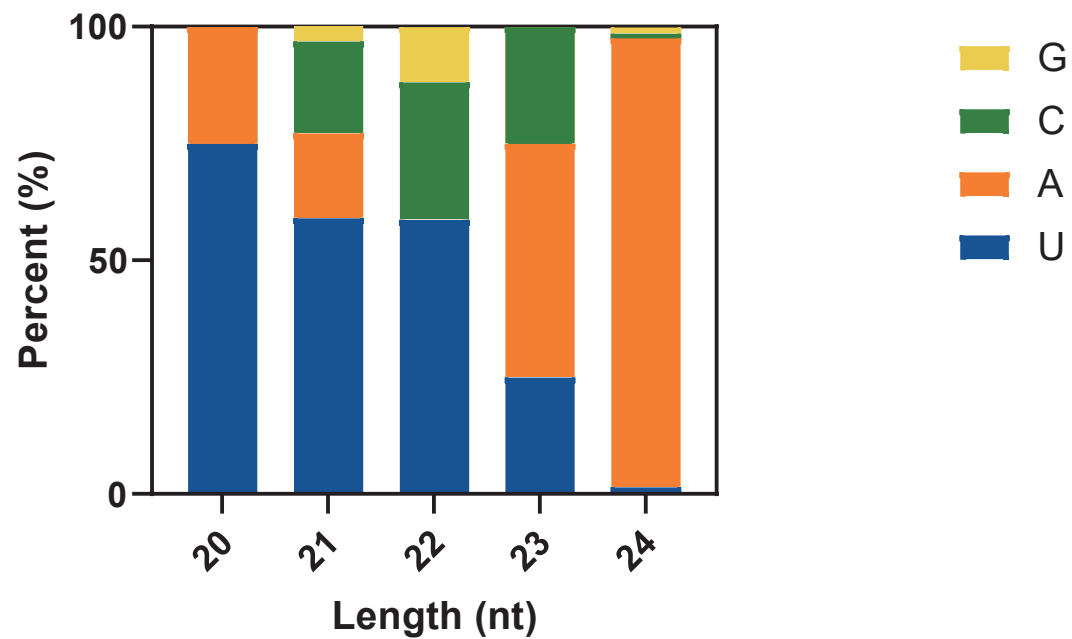

Supplement: Web_Material_uhae094 [file web_material_uhae094.zip › Figure S1.pdf]

**Mdo-miR482a**

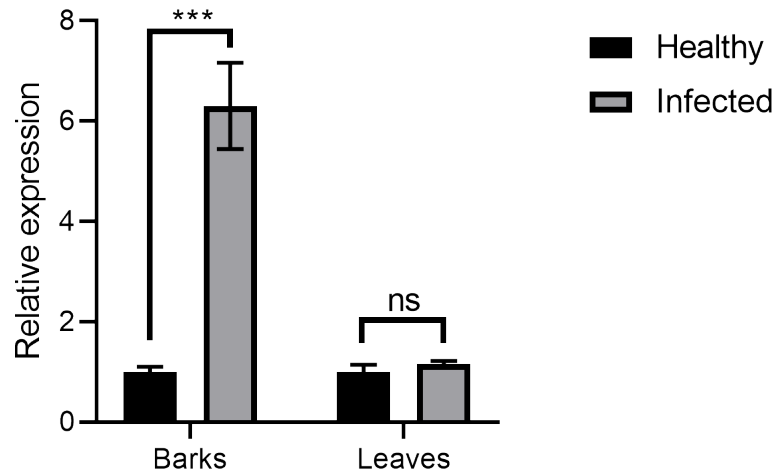

**Mdo-miR482b**

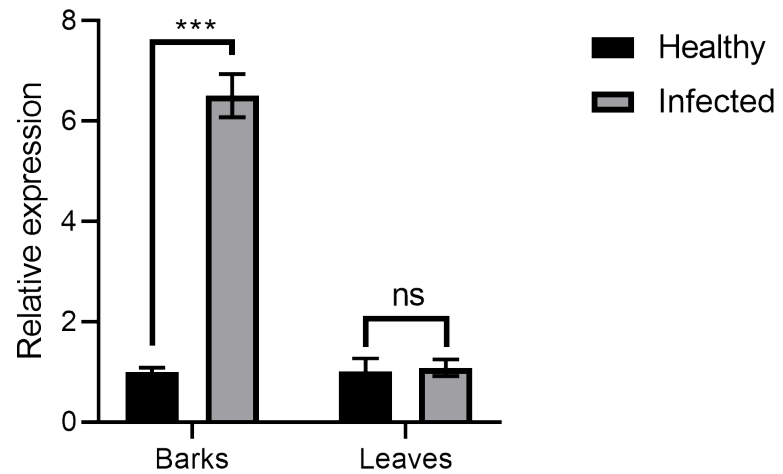

Supplement: Web_Material_uhae094 [file web_material_uhae094.zip › Figure S2.pdf]

**MD03G1071300**

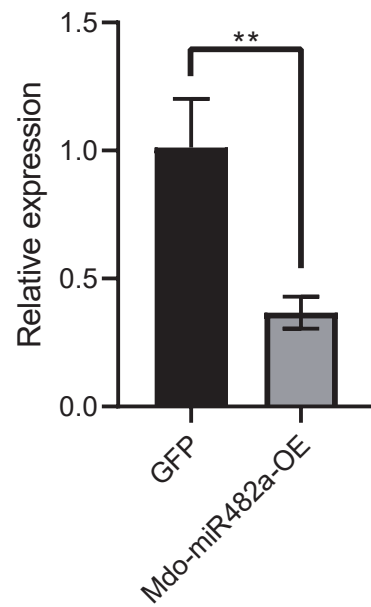

**MD15G1042600**

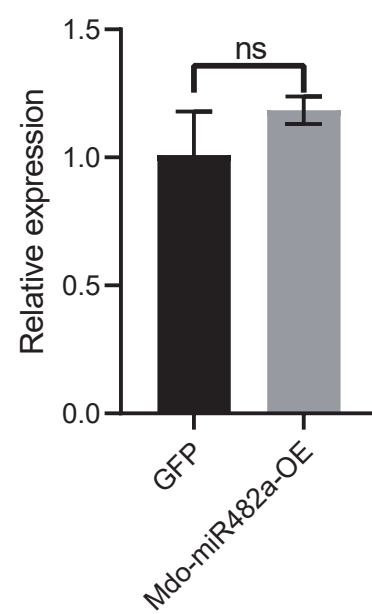

**MD04G1238000**

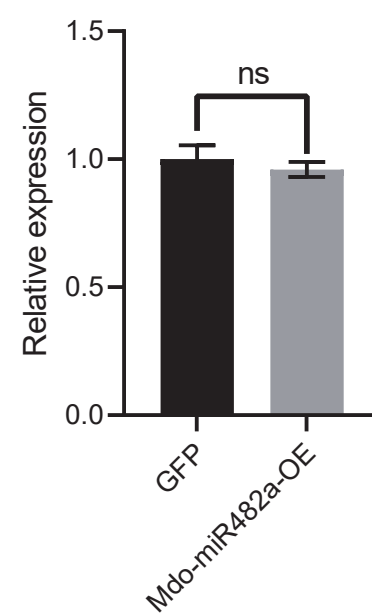

**MD06G1024500**

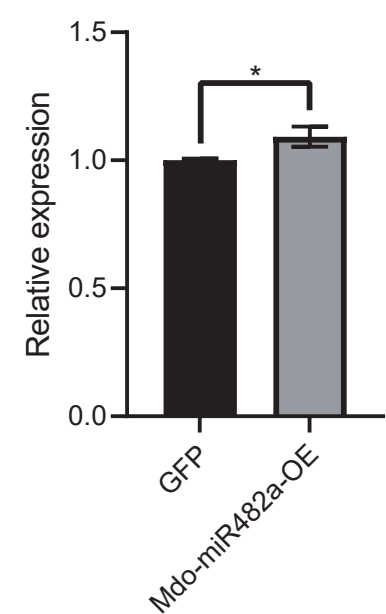

**MD08G1104200**

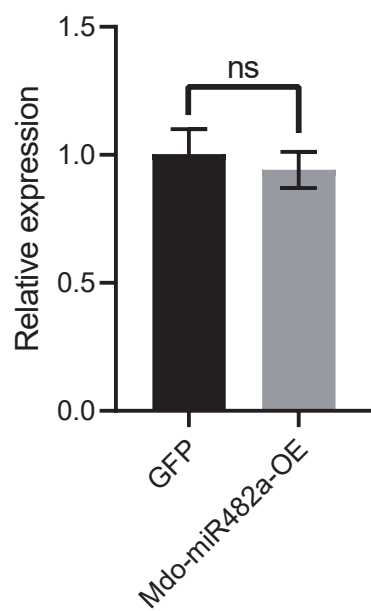

**MD08G1099900**

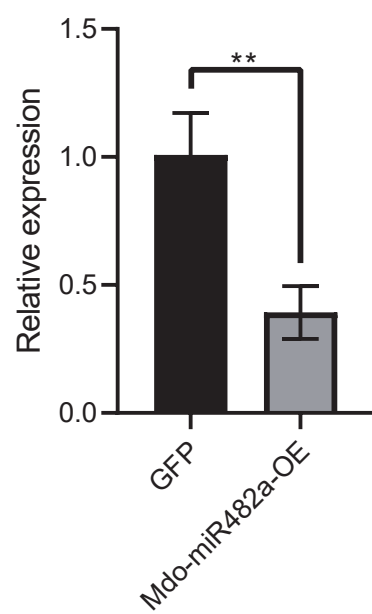

**MD12G1256300**

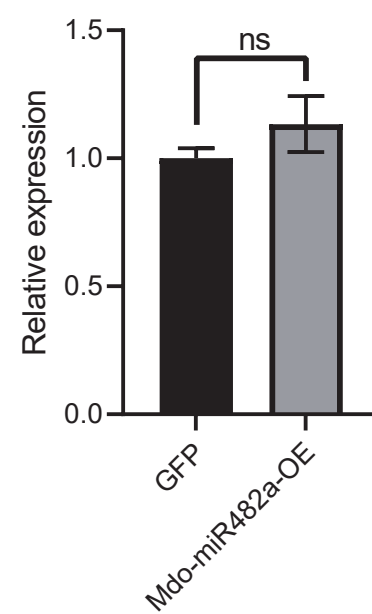

**MD08G1093300**

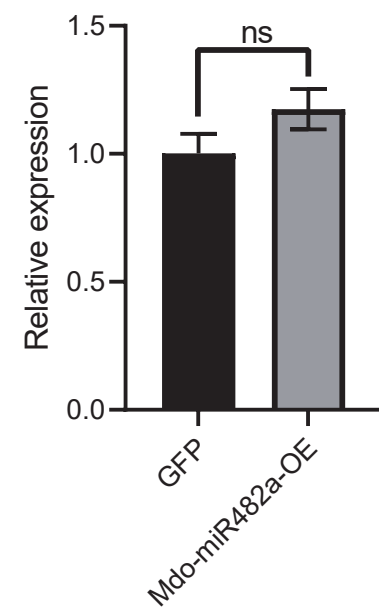

Supplement: Web_Material_uhae094 [file web_material_uhae094.zip › Figure S3.pdf]

**A**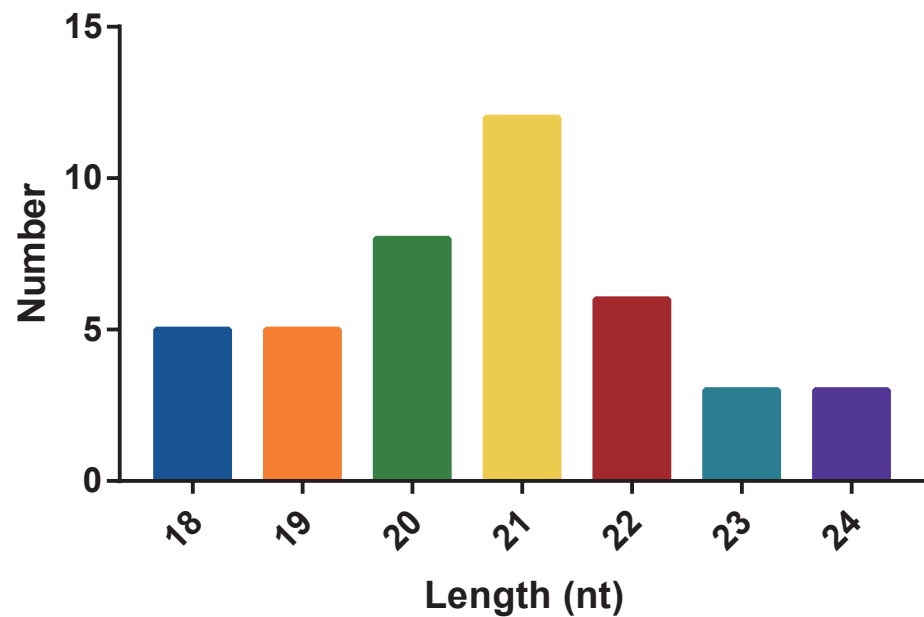**B**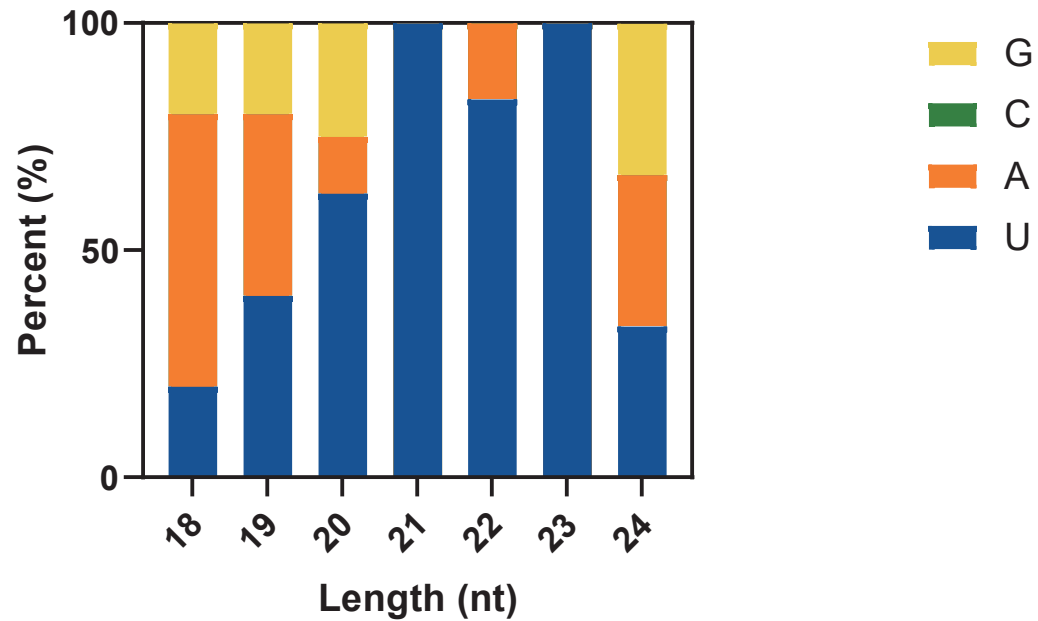

Supplement: Web_Material_uhae094 [file web_material_uhae094.zip › Figure S4.pdf]

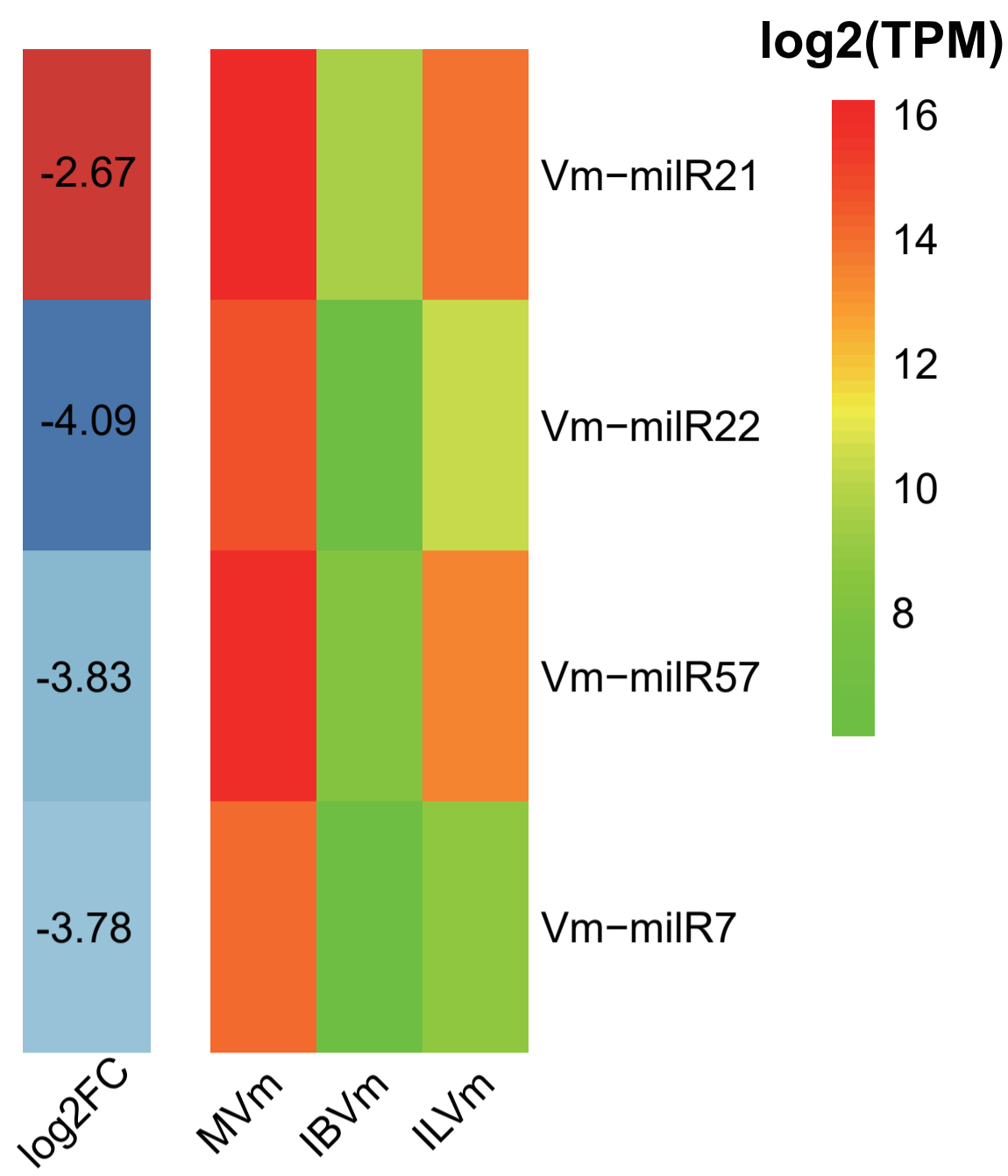

Supplement: Web_Material_uhae094 [file web_material_uhae094.zip › Figure S5.pdf]

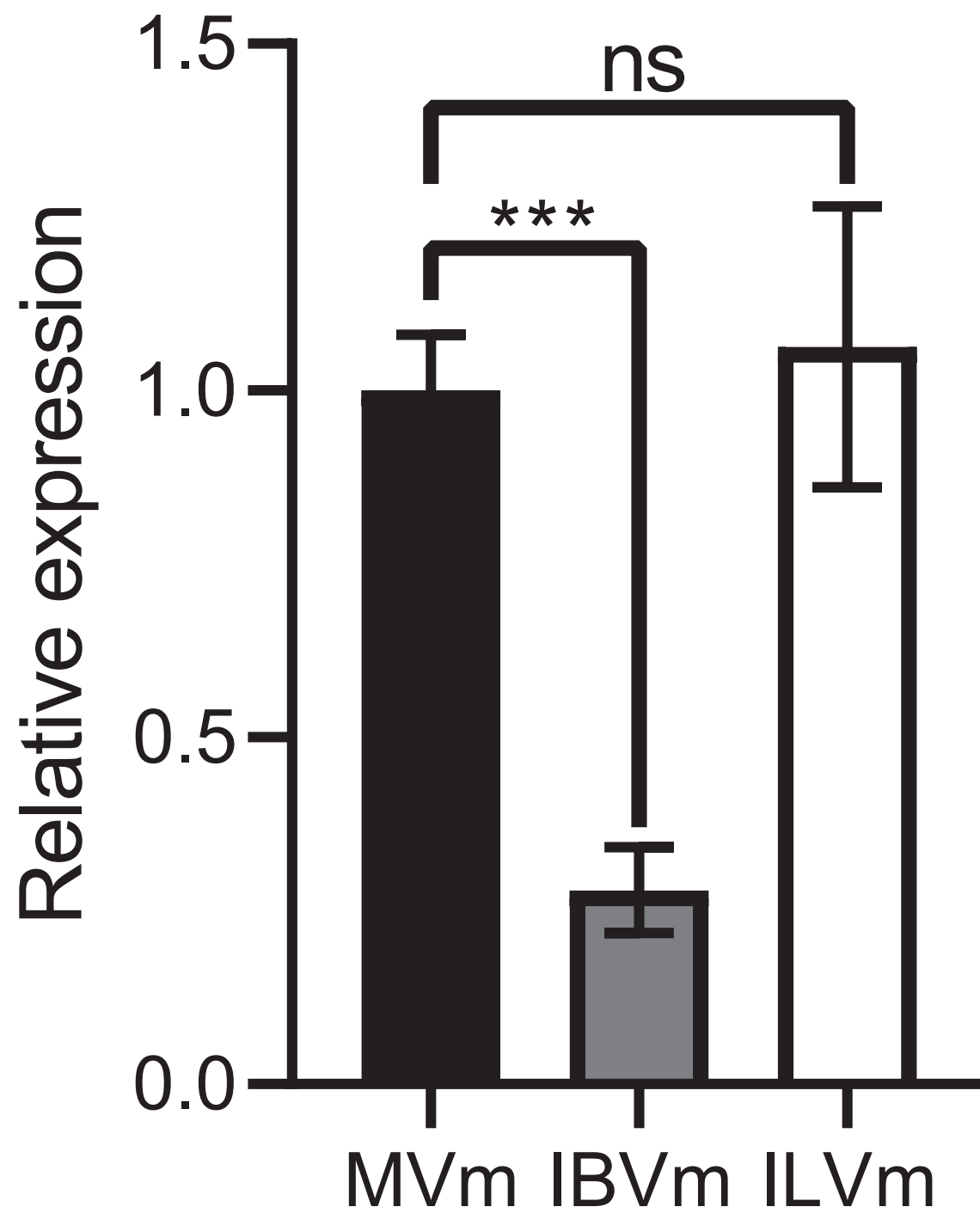

Supplement: Web_Material_uhae094 [file web_material_uhae094.zip › Figure S6.pdf]

**A**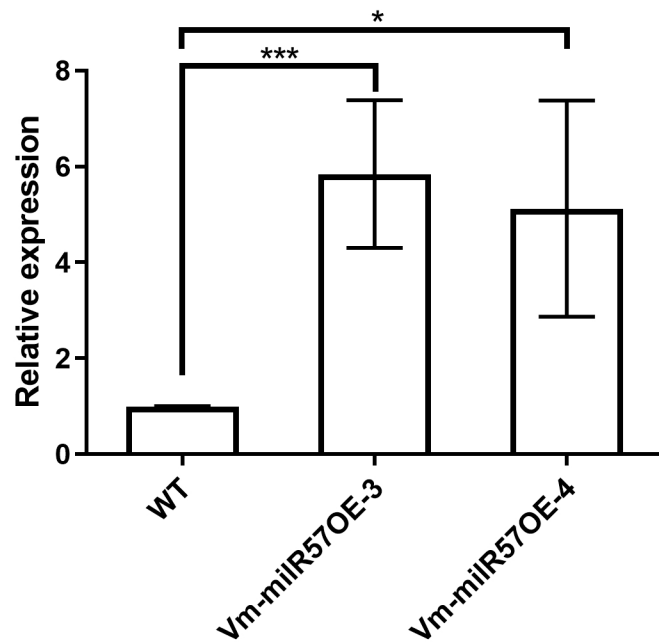**B**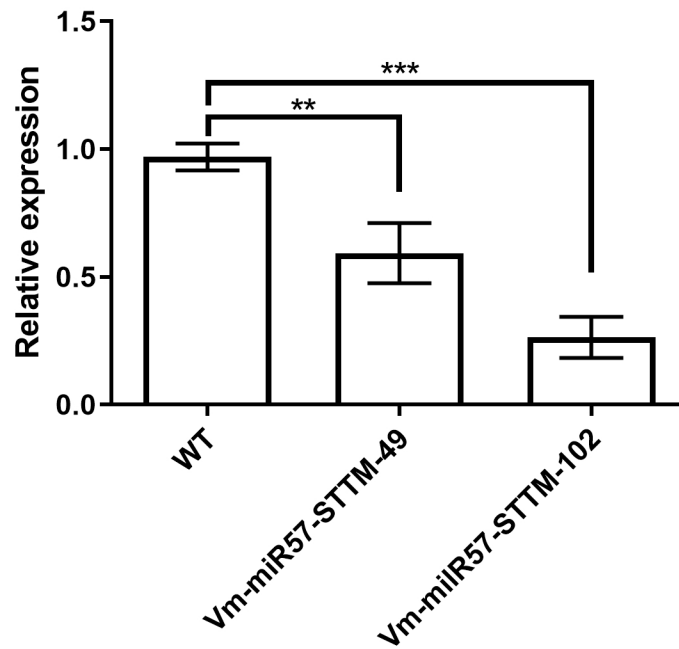

Supplement: Web_Material_uhae094 [file web_material_uhae094.zip › Figure S7.pdf]

A

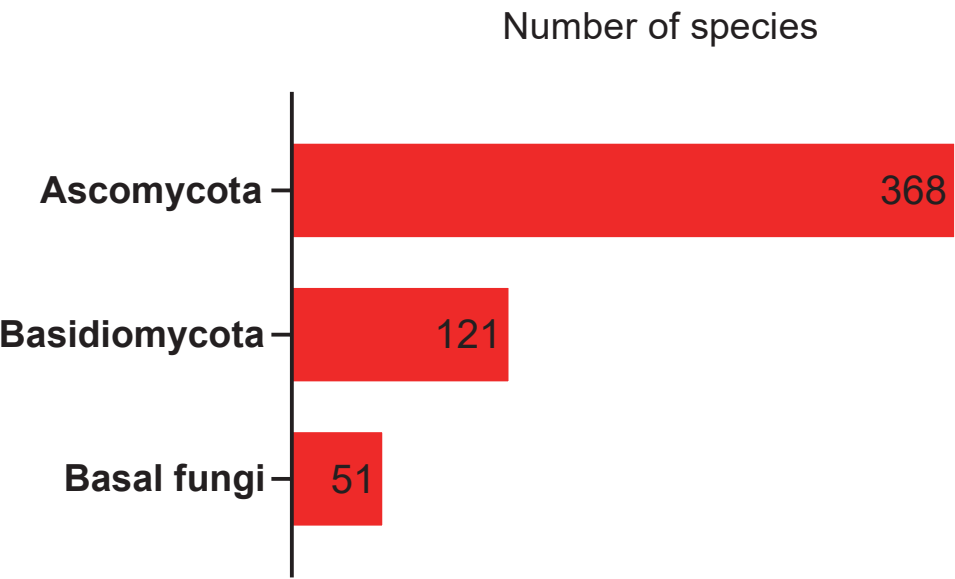

B

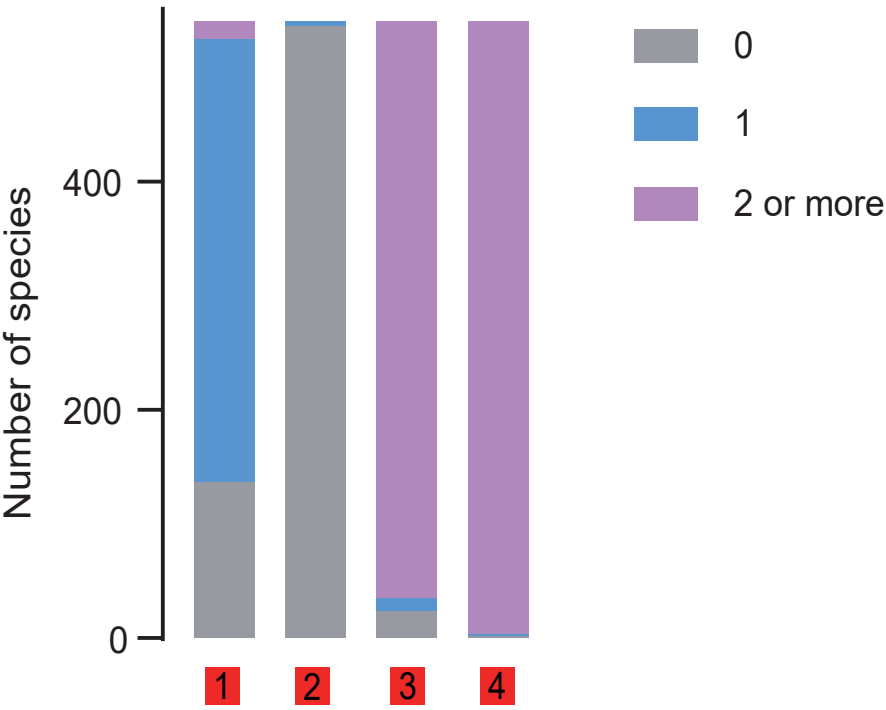

C

- Presence  
● Absence

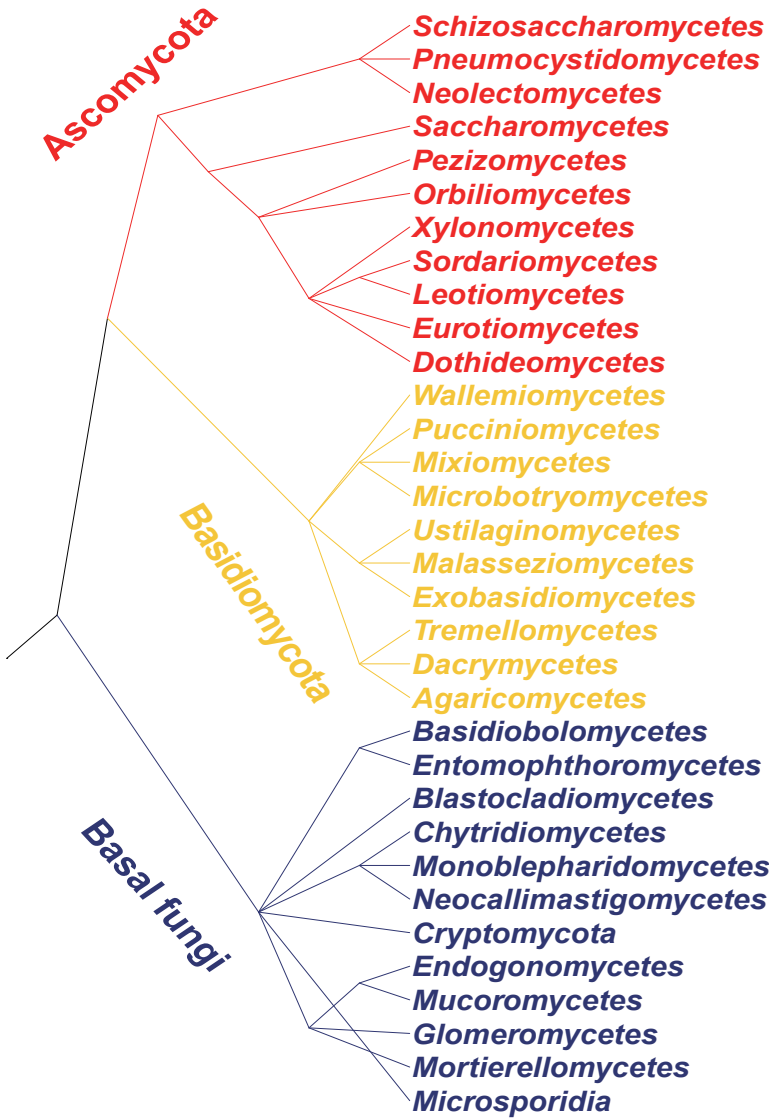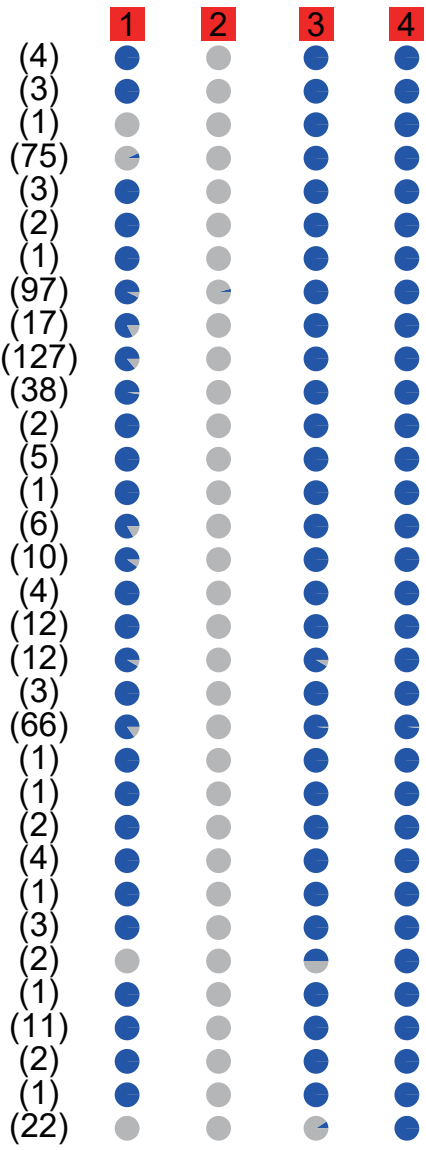

Supplement: Web_Material_uhae094 [file web_material_uhae094.zip › Figure S8.pdf]

**A**

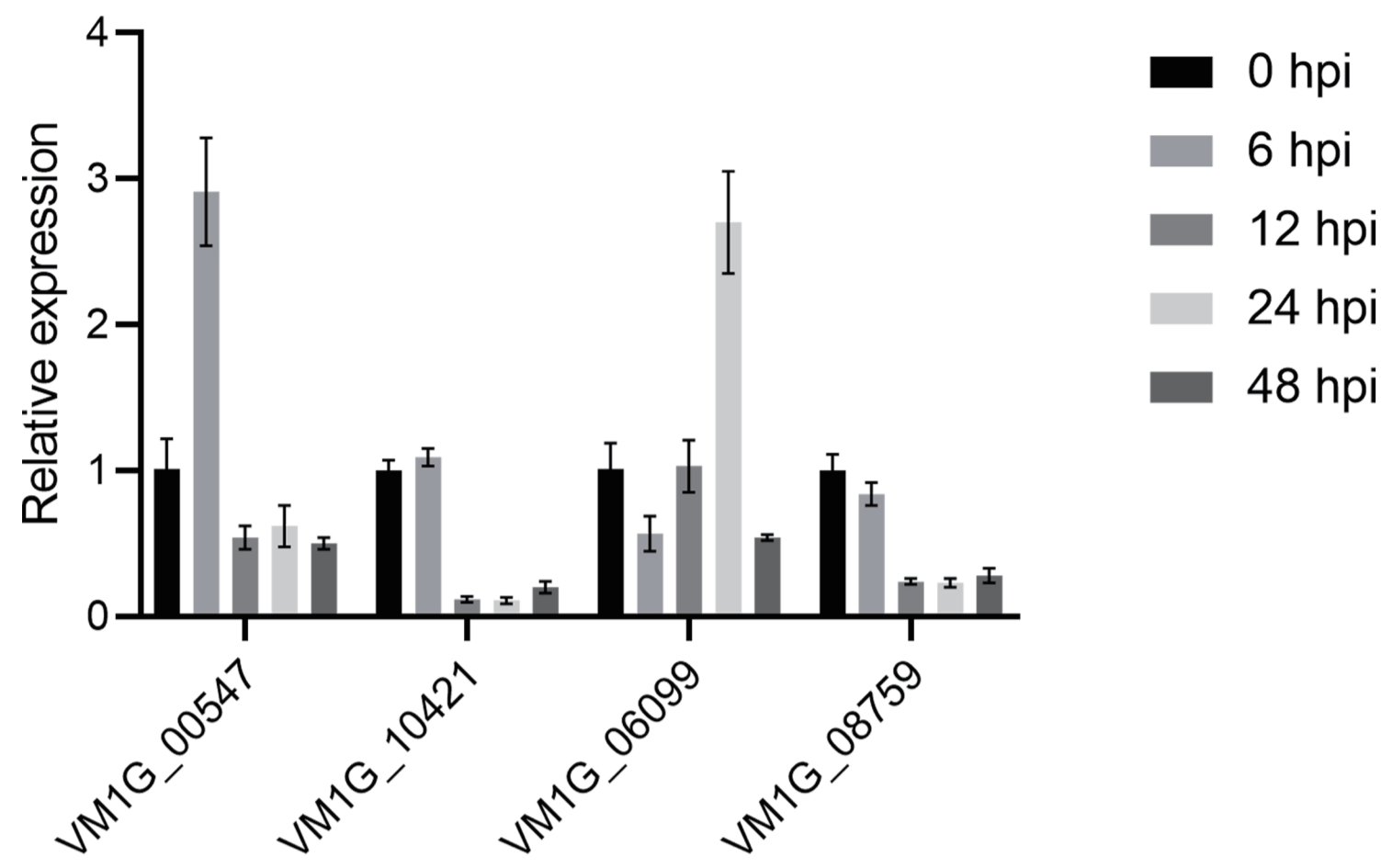

**B**

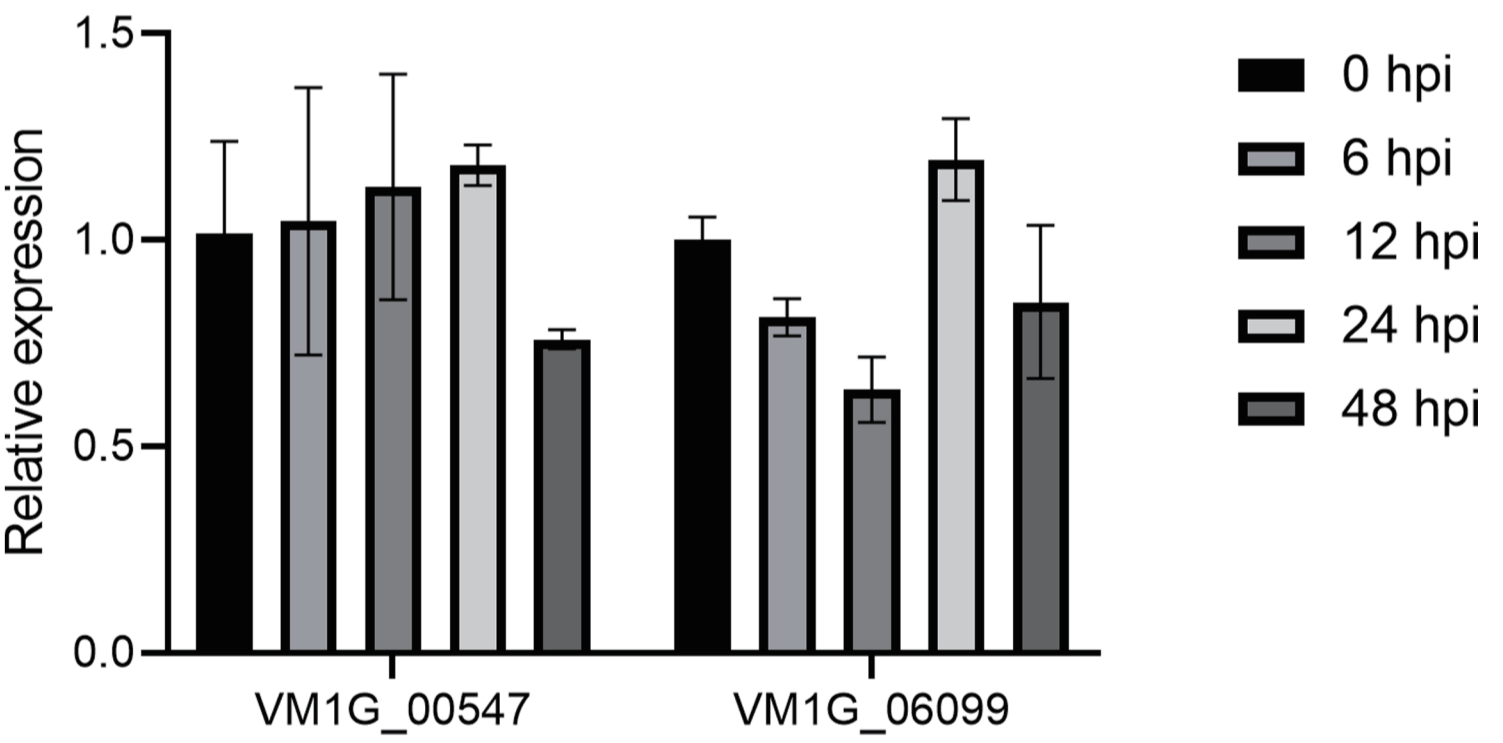

Supplement: Web_Material_uhae094 [file web_material_uhae094.zip › Figure S9.pdf]

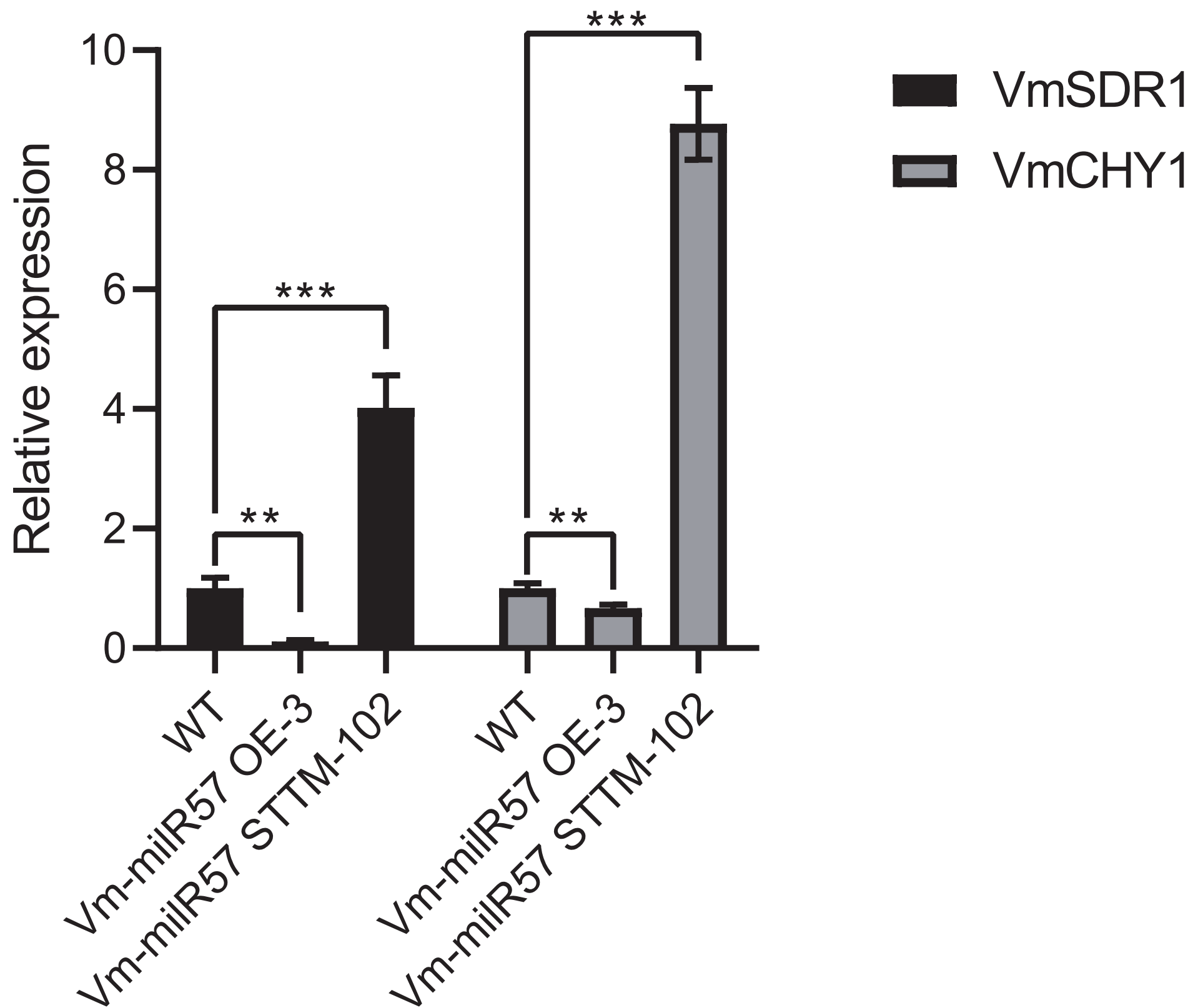

Supplement: Web_Material_uhae094 [file web_material_uhae094.zip › Figure S10.pdf]

|                  |   |   |   |   |
|------------------|---|---|---|---|
| <i>Vm-milR57</i> | - | - | + | + |
| VmSDR1           | + | - | + | - |
| VmSDR1-m         | - | + | - | + |

*Vm-milR57*

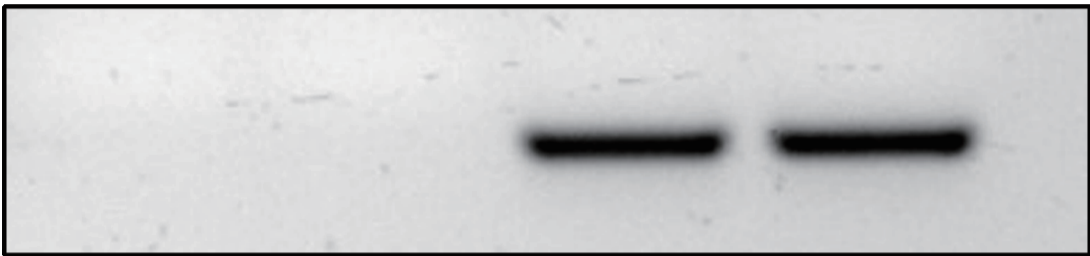

rRNA

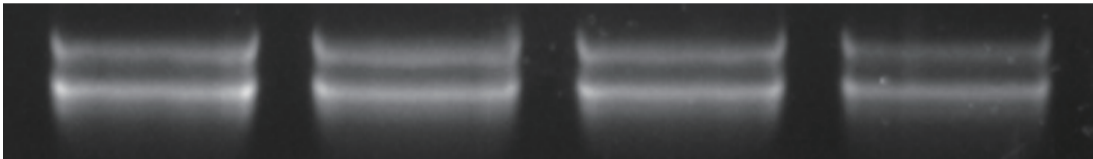

Supplement: Web_Material_uhae094 [file web_material_uhae094.zip › Figure S11.pdf]

*ΔVmSDR1-7*

*ΔVmSDR-12*

*ΔVmCHY1-5*

*ΔVmCHY-149*

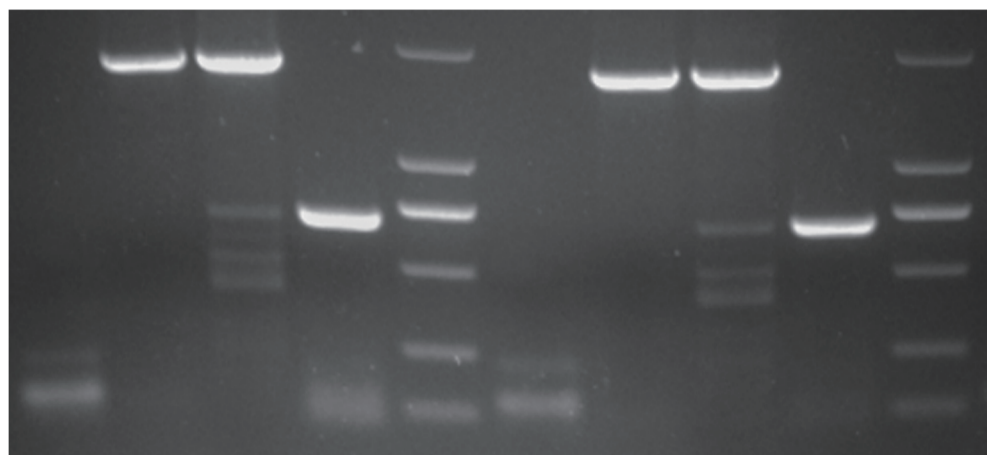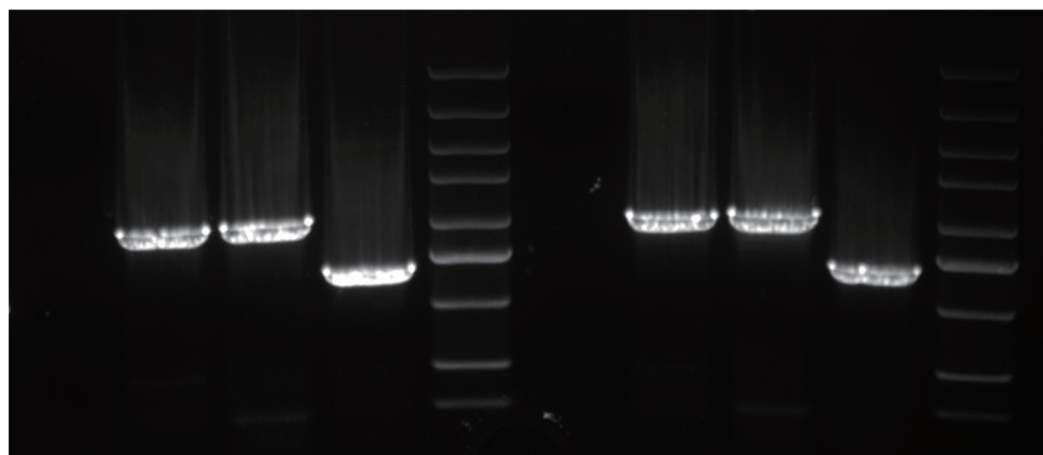

Supplement: Web_Material_uhae094 [file web_material_uhae094.zip › Figure S12.pdf]

**A**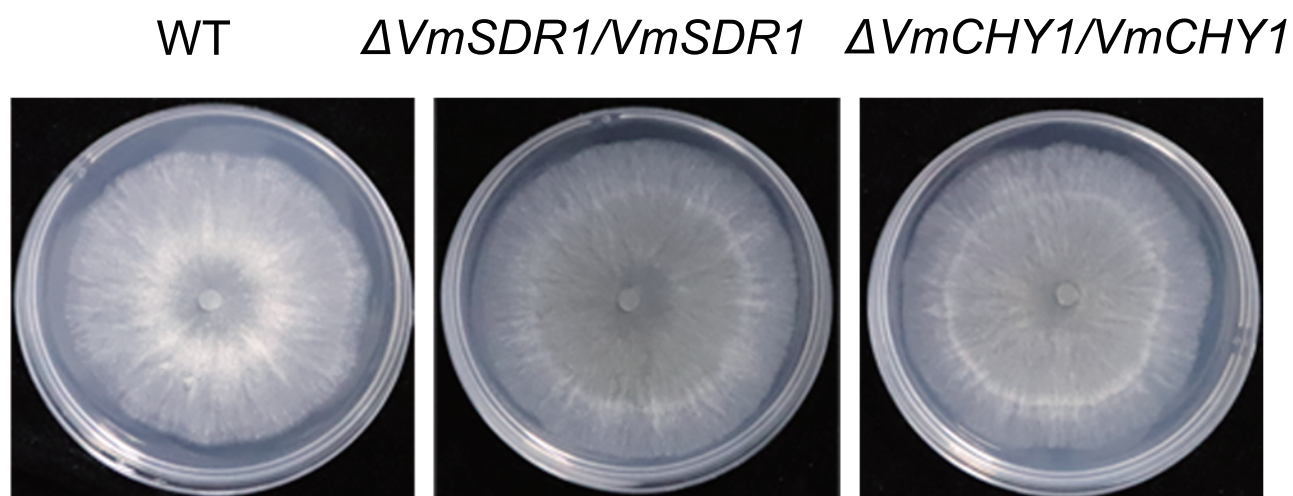**B**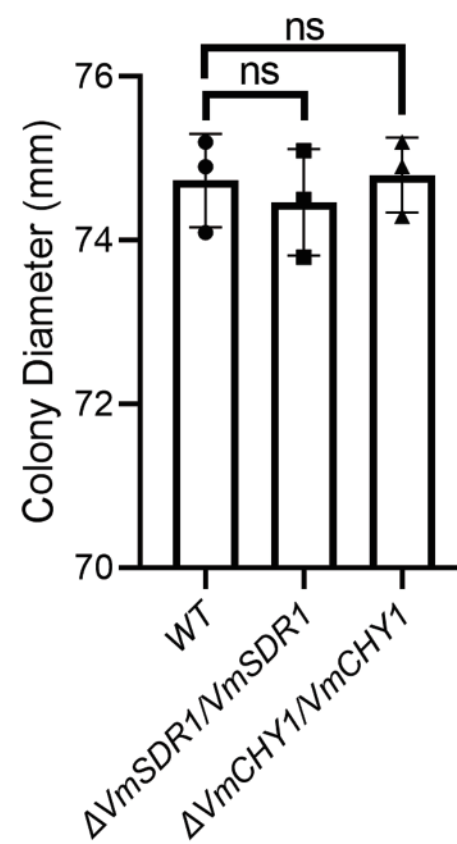**C**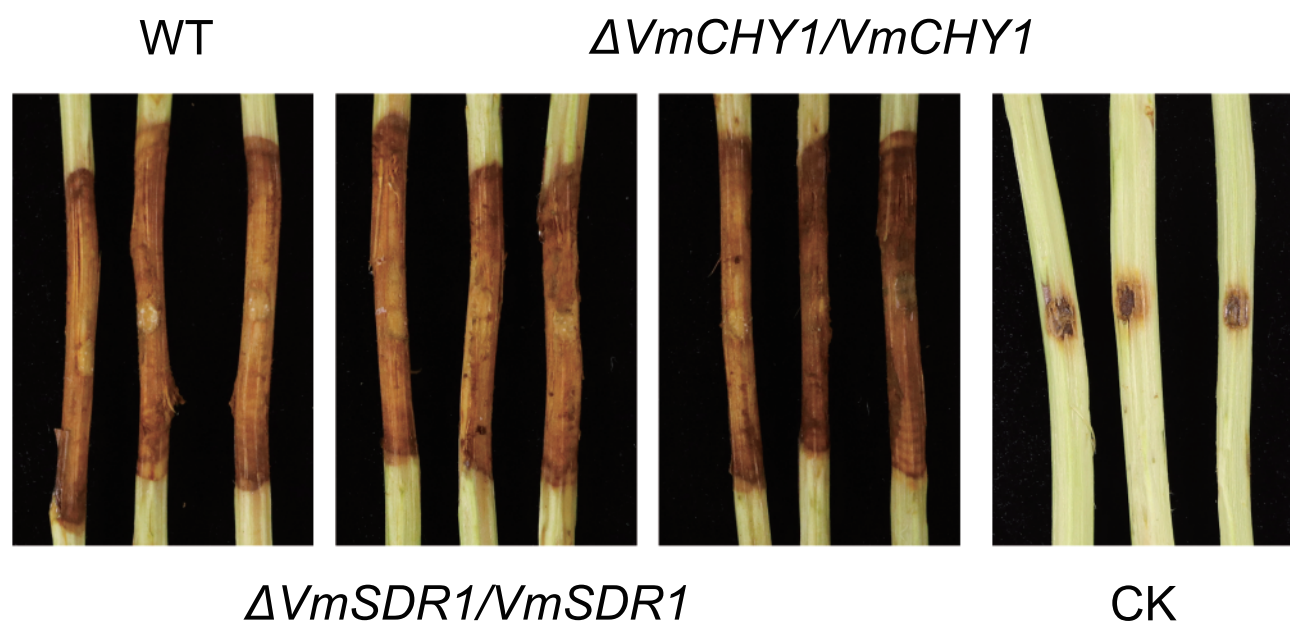**D**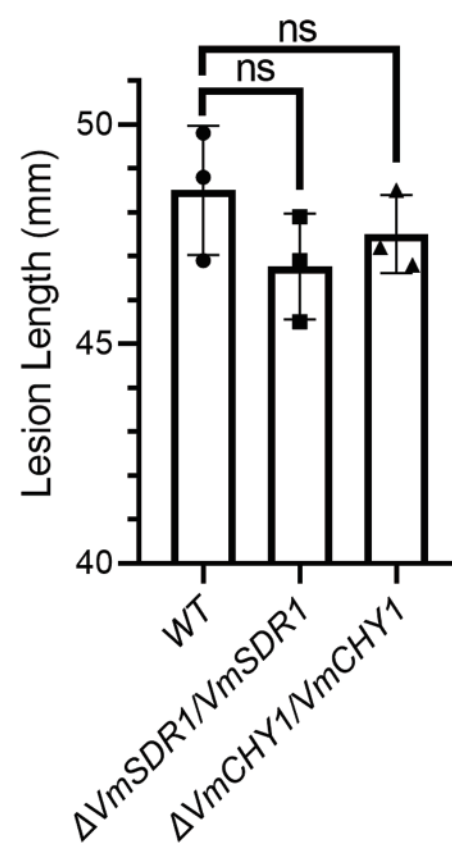**E**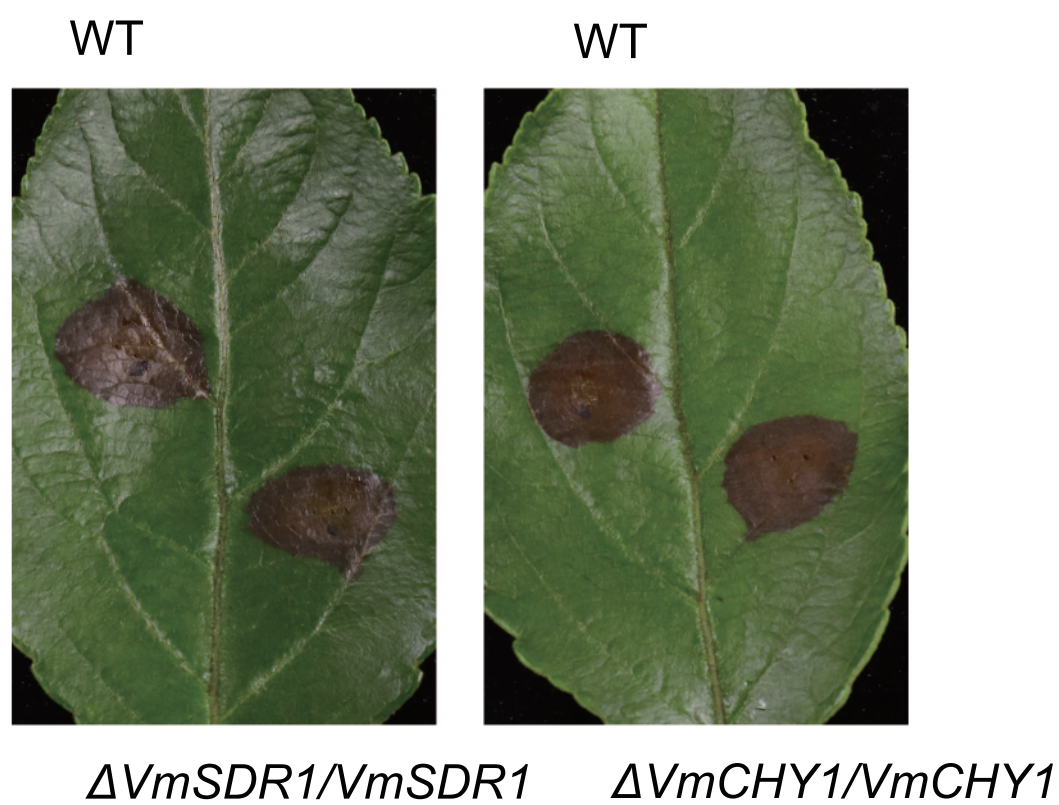**F**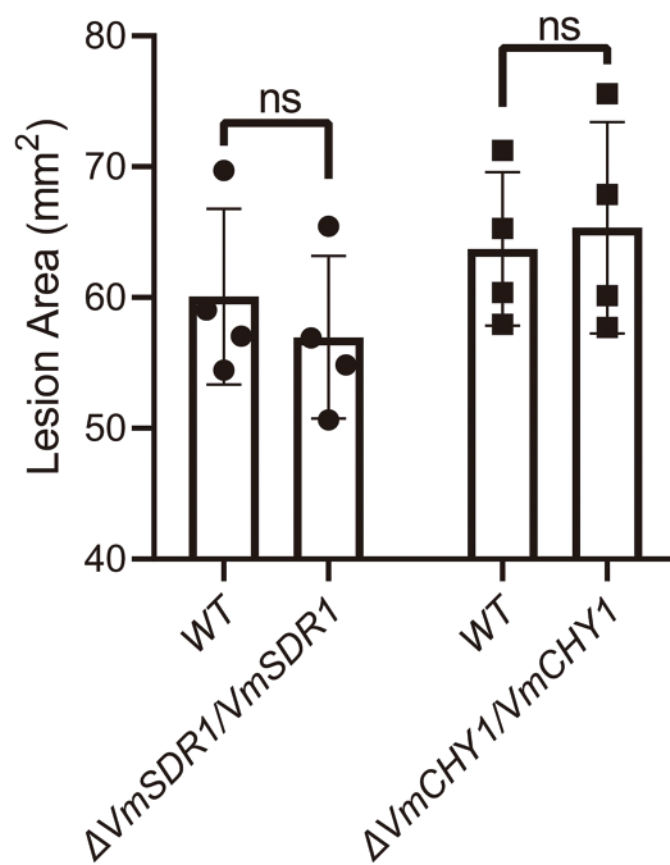

Supplement: Web_Material_uhae094 [file web_material_uhae094.zip › Figure S13.pdf]
